# Fine Motor Skills Predict Maths Ability Better than They Predict Reading Ability in the Early Primary School Years

**DOI:** 10.3389/fpsyg.2016.00783

**Published:** 2016-05-30

**Authors:** Nicola J. Pitchford, Chiara Papini, Laura A. Outhwaite, Anthea Gulliford

**Affiliations:** School of Psychology, University of NottinghamNottingham, UK

**Keywords:** fine motor skills, literacy, maths, executive functions, socio-economic status, early years education

## Abstract

Fine motor skills have long been recognized as an important foundation for development in other domains. However, more precise insights into the role of fine motor skills, and their relationships to other skills in mediating early educational achievements, are needed to support the development of optimal educational interventions. We explored concurrent relationships between two components of fine motor skills, Fine Motor Precision and Fine Motor Integration, and early reading and maths development in two studies with primary school children of low-to-mid socio-economic status in the UK. Two key findings were revealed. First, despite being in the first 2 years of primary school education, significantly better performance was found in reading compared to maths across both studies. This may reflect the protective effects of recent national-level interventions to promote early literacy skills in young children in the UK that have not been similarly promoted for maths. Second, fine motor skills were a better predictor of early maths ability than they were of early reading ability. Hierarchical multiple regression revealed that fine motor skills did not significantly predict reading ability when verbal short-term memory was taken into account. In contrast, Fine Motor Integration remained a significant predictor of maths ability, even after the influence of non-verbal IQ had been accounted for. These results suggest that fine motor skills should have a pivotal role in educational interventions designed to support the development of early mathematical skills.

## Introduction

Converging evidence from neuroimaging studies, brain-lesioned patients, and developmental disorders suggests a fundamental interrelation between motor and cognitive development (see Diamond, 2000, for a review). For example, brain imaging studies have demonstrated a strong functional coupling between brain regions typically thought to underpin exclusively either cognitive or motor processes (Abe and Hanakawa, 2009; Stoodley, 2012). In addition, clinical populations, such as those identified with Attention Deficit Hyperactivity Disorder and Development Coordination Disorder, that were originally associated with a single domain now show notable co-occurrence of both motor and cognitive difficulties (Piek et al., 1999; Pitcher et al., 2003; Alloway, 2007).

Evidence is also accumulating from studies of typically developing children for a close association between motor and cognitive development. When gross measures of motor and cognitive skills are considered, results from across several studies produce inconsistent findings as to the extent and significance of the relationship between motor and cognitive development (e.g., Wassenberg et al., 2005; Roebers and Kauer, 2009; Davis et al., 2011; Jenni et al., 2013). However, when motor and cognitive skills are subdivided into different components, specific correlations emerge. A recent review by van der Fels et al. (2014) systematically investigated findings from typically developing children aged 4–16 years. The authors concluded that the link between motor and cognitive domains could be explained more specifically by relationships between fine motor skills and higher-order cognitive skills. This corroborates the results of Davis et al. (2011), who found that fine motor skills and visual attention underpinned the more generic association between motor and cognitive domains.

Different definitions and operationalizations of fine motor skills are apparent in the literature, but we consider fine motor skills to “encompass control and coordination of the distal musculature of the hands and fingers,” as defined by Bruininks and Bruininks (2005, p. 2). Within this definition two different components are distinguished: (1) Fine motor integration is conceptualized as a manual ability which requires synchronized hand–eye movements and the processing of a visual stimulus in order to produce adequate motor output; and (2) fine motor precision is conceptualized as a ‘pure’ fine manual skill which relies on a minimal visual-perceptual component. Fine motor integration, but not fine motor precision, has been shown to contribute significantly to academic achievement (Carlson et al., 2013), suggesting these are separate components of fine motor skills.

Fine motor skills are thought to be essential for early learning. On average, in regular kindergarten schools in the US 33–66% of daily activities involve fine motor skills, such as coloring, copying, cutting, and drawing (Marr et al., 2003). A similar percentage of time has been recorded in US primary schools as being dedicated to activities that involve fine motor skills (McHale and Cermak, 1992). Fine motor skills have been shown to be a powerful predictor of school readiness (Grissmer et al., 2010) and fine, but not gross, motor skills are sometimes included in assessments of school readiness (Bala et al., 2010). In addition, fine motor skills are related to school adaptation and social behavior during the transition from preschool to primary school (Bart et al., 2007) and classroom engagement at the end of second grade (Pagani et al., 2010). These studies highlight the integral relationship that development of early fine motor skills has with readiness and adaptation to early primary school.

Fine motor skills in the early years have also been shown to predict later academic achievement, especially in reading and mathematics (e.g., Son and Meisels, 2006; Grissmer et al., 2010; Pagani et al., 2010; Cameron et al., 2012; Dinehart and Manfra, 2013) and to predict underachievement in able students at school (Stoeger et al., 2008, 2013). In particular, fine motor integration has received more attention than tasks of fine motor precision and fine motor integration has been identified to be a strong predictor of later achievement (Tramontana et al., 1988; Kulp, 1999; Kurdek and Sinclair, 2001).

Possible mechanisms underpinning the link between fine motor skills and scholastic attainment have recently been put forward. Cameron et al. (2016), for example, highlighted that fine motor skills afford children the opportunity to practice mapping visual representations to emerging literacy and mathematical skills, through practicing writing letters, counting objects, and sorting objects into similar categories based on mathematical concepts, such as number (e.g., groups of three), shape (e.g., squares and circles), and size (e.g., big and small). Likewise, Becker et al. (2014) argued that good fine motor skills enables children to script letters and numbers automatically and this in turn directs cognitive resources toward conceptual processes, such as connecting figures and sounds, understanding mathematical concepts and decoding words.

Accordingly, for maths, fine motor skills may underlie the acquisition of quantitative and spatial concepts and could be supported through early years classroom activities and aids that capitalize on fine motor skills for successful execution, such as Snap Cubes, Numicon, and cutting out shapes. In contrast, the link between fine manual skills and reading acquisition is likely to emerge through writing, when children begin to represent letters and words on a page. The phonics instruction that is taught in UK primary schools as standard (Department for Education, 2010) also makes use of fine motor skills to assist the mapping of sounds through actions (e.g., running fingers up the arm to represent ants whilst saying the sound /a/). However, it seems that precise motor movements are not as critical in supporting early phonological skills through sound-based interventions as they might be for acquiring early mathematical skills, where the motor movement maps directly to the conceptual representation. A specific illustration of this is with finger counting which requires direct linkage between precise finger movements and the corresponding number concept. Whilst there has been much focus on the role of role of finger counting in mathematical ability, the most recent consensus appears to be that fingers can assist but are not necessary in the acquisition of number skills (e.g., Crollen et al., 2011a,b; Lafay et al., 2013). As children learn to write letters and numbers over the first year of school, the relationship between fine motor skills and reading and maths ability may become stronger over this period, when precision in scripting letters and numbers is necessary in supporting the mappings between letters to sounds and words and numbers to numerical concepts.

In addition, a specific role of visuo-spatial skills and fine motor precision has been proposed to underpin some mathematical abilities. For example, Barnhardt et al. (2005) showed that children with poor visual motor integration skills made more errors in spacing letters, words, and number problems on a page relative to their peers with good visual motor integration skills. They argued that difficulties with spatial alignment and organization of letters and numbers on a page can lead to incorrect answers in maths tasks, even when the underlying computation might be accurate. Similarly, in a recent study with UK children aged 8–10 years, Simms et al. (2016) showed that tasks of visuo-motor integration and visuo-spatial skill were significantly related to performance on the number line estimation task, which is purported to measure precise numerical representations (e.g., Siegler and Ramani, 2008) and proportional judgment skills (e.g., Ebersbach et al., 2008). They suggested that the spatial components and need for motor precision in the number line estimation task accounted for the observed association with visuo-motor integration and visuo-spatial skills. This suggests there might be a specific role for fine motor precision in early mathematical attainment, yet to date, no study has attempted to differentiate this from fine motor integration skills.

Fine motor skills have been associated with several other cognitive abilities, including processing speed (Wassenberg et al., 2005), executive functions (Livesey et al., 2006; Rigoli et al., 2012), and scholastic skills (Morales et al., 2011). Recent studies that have investigated how fine motor skills are associated with literacy and maths abilities have also recognized the importance of executive functions in mediating this relationship. For example, in a prospective study spanning the transition into kindergarten, Cameron et al. (2012) found specific effects of fine motor integration (using the design copy task from the Early Screening Inventory-Revised, Meisels et al., 1997) and executive functions on six measures of achievement, including literacy and maths, as assessed with the Woodcock–Johnson III Tests of Achievement (Woodcock et al., 2001). Becker et al. (2014), in a concurrent study with 127 pre-kindergarten and kindergarten children in the US, finding that visual-motor skills and behavioral self-regulation significantly predicted early maths ability and emergent literacy, noted that executive functions were additionally related to early literacy skills, In contrast, in a concurrent study with a sample of 97 pupils spanning 5–18 years, Carlson et al. (2013) reported a specific role for fine motor integration but not fine motor coordination in predicting mathematics ability; but neither components of fine manual control contributed significantly to reading ability when SES, gender and IQ were controlled. Further still, a longitudinal study of children in early primary school by Roebers et al. (2014) failed to find a specific role for fine motor skills and non-verbal IQ in predicting mathematics, reading and spelling ability, once executive functions were accounted for.

These studies imply a complex relationship between the development of fine motor, cognitive and scholastic skills. However, other demographic characteristics, such as gender and socio-economic status (SES), are known to affect these abilities. Gender differences have been reported in the development of fine compared to gross motor skills (e.g., Sigmundsson and Rostoft, 2003). A recent study by Morley et al. (2015) conducted in the UK with 4- to 7-year-old children showed that, after controlling for age, girls outperformed boys on all tasks involving fine motor skill, whereas boys outperformed girls on gross motor tasks involving catching and dribbling a ball. Furthermore, a study by McPhillips and Jordan-Black (2007) compared motor development in two groups of children, aged 4–5 years and 7–8 years, from two schools of low SES and two schools of high SES in Northern Ireland. For both age groups, they found a significant main effect of SES and gender on the overall motor score but no interaction effect. Specifically, on tasks of manual dexterity, they found that girls outperformed boys and children of lower SES performed less well than those of higher SES.

The evidence accrued in the literature thus far implies that underdevelopment of fine motor skills in the early years might be a significant risk factor for later scholastic attainment. Children from low socio-economic backgrounds are vulnerable to poor development of fine motor skills in the early years (Dinehart and Manfra, 2013; Morley et al., 2015) and there has been some consideration of the environmental factors influencing this relationship (Vazir et al., 1998; de Barros et al., 2003), with the lack of opportunities to engage with preschool activities that promote fine motor skills being a prominent explanation. SES is also known to predict early cognitive development (e.g., Duncan et al., 1994), the development of executive functions, i.e., the ability to follow commands, remember sequences of information and inhibit responses, and the development of these underlying skills in early scholastic skills (e.g., Duncan et al., 2007; Mazzocco and Kover, 2007).

Understanding the impact of gender and SES on fine motor skills is particularly relevant in the UK, where children from low SES backgrounds have been identified to be at risk of lower reading and maths skills compared to higher SES populations (Strand, 1997, 2014; Sammons et al., 2004; Anders et al., 2012). More specifically, boys from white working class backgrounds are purported to be of particular risk to underachievement at school (Strand, 2014). This is particularly problematic to Nottinghamshire, the region where this study took place, because pockets of underachievement in children from low SES homes are present across the shire.^1^ Achievement in basic skills of literacy and numeracy is a question of significant concern in the UK. These long-held concerns focus upon a proportion of lower attaining students, resistant to overall rises evident in national attainment data sets, a so-called ‘stubborn-tail of underachievement’ (Tymms and Merrell, 2007). In order to address underachievement in reading attainments, the UK Government has implemented a phonics-based reading intervention across all primary schools (Department for Education, 2010). In contrast, there is currently no specified national intervention for maths.

The inception of this study came through a request from head teachers within this region, who had grown increasingly concerned about the role of fine motor skills in the development of reading and maths skills in the early primary years, perceiving that upon entering the school system at age 4 years, children from low socio-economic backgrounds were apparently showing impoverished manual skills. It was thus important to determine the contributory effects of fine motor skills on early reading and maths performance in boys and girls from low–medium SES backgrounds in the first years of primary school in order to target interventions most appropriately. In this study, we explore the relationship between fine motor skills and scholastic performance in the early primary school years (Study 1) whilst taking into account other potential influences on performance (Study 2). Considering educational programs aimed at raising achievement, we additionally viewed these investigations as an opportunity to explore the relative effects of the UK’s national literacy intervention, on the one hand, and in contrast, the absence of a national strategy to support numeracy acquisition, on the other. The questions raised by the existing literature coincided with head teacher concerns: both were therefore investigated through an exploration of concurrent associations between fine motor skills and scholastic abilities, in two samples of primary school children.

To date, no study has investigated concurrently the role of fine motor skills on the development of early scholastic skills across genders, in a UK sample of low–medium SES pupils in the early primary school years. Most studies reported in the literature are prospective and investigate the influence of fine motor skills in preschool years on later acquired scholastic skills (Son and Meisels, 2006; Luo et al., 2007; Grissmer et al., 2010; Pagani et al., 2010; Cameron et al., 2012; Dinehart and Manfra, 2013; Roebers et al., 2014). Concurrent investigation of the association between fine motor skills and early scholastic skills, taking into account SES and gender, will provide insight into how these factors are related at a particular age. This is important for informing interventions that may optimally support early years development in literacy and numeracy, where emerging evidence suggests they may require a component of fine motor skill. Studies that have adopted concurrent investigation of fine motor skills and scholastic abilities tend to span a broad age range and do not focus on the early primary years (e.g., Carlson et al., 2013). In addition, they tend to focus on just one aspect of fine motor skill, such as fine motor integration (e.g., Kulp, 1999; Becker et al., 2014; Santi et al., 2015). To understand more precisely the role of fine motor skills in early reading and maths acquisition different components of fine manual control need to be assessed (Grissmer et al., 2010; Cameron et al., 2012; Carlson et al., 2013). In addition, the contribution of reading on early maths ability and vice versa needs to be examined in relation to fine motor skills, as Duncan et al. (2007) found early reading and mathematical skills were the strongest predictors of both reading and mathematics in middle childhood. Likewise, Hooper et al. (2010) found similar results across different ethnic groups and Purpura et al. (2011) found three measures of early literacy to be predictive of both concurrent and subsequent mathematical ability. This association may result from co-dependence of similar skills, so considering the influence of fine motor skills on reading and maths ability, whilst taking into account concurrent maths and reading ability, respectively, controls for the potential impact of additional skills that are related to both reading and maths. To our knowledge, to date, no study has investigated concurrent relationships between early reading and maths ability whilst simultaneously investigating the influence of different components of fine motor skill.

The present study investigated the role of two components of fine manual control in the development of early reading and maths skills. Measures of Fine Motor Integration and Fine Motor Precision were taken from the same assessment battery of motor development (BOT-2, Bruininks and Bruininks, 2005) enabling direct comparison to be made between these two components of fine manual control. Similarly, measures of Word Reading and Mathematical Reasoning were taken from the Weschler Individual Achievement Test second Edition (Wechsler, 2005) enabling direct comparison to be made between these key scholastic skills. Our focus was on pupils from low SES backgrounds to address the high levels of underachievement that is commonly reported in this population. In study 1, we explored concurrent relationships between fine motor skills and reading and maths ability in a group of children aged 5–7 years from low SES backgrounds attending Year 1 of primary school. In study 2, we explored the interrelation of non-verbal IQ and verbal short-term memory with fine motor skills and reading and maths ability in a group of Foundation year children aged 4–5 years from low-to-medium SES backgrounds. Verbal short-term memory was used in the study as a proxy for working memory, which is considered to be a measure of executive functions. Complex span tasks are typically used to assess working memory but are difficult for young children to perform reliably, as working memory starts to develop from about 4 years of age (Gathercole et al., 2004). We used a simple span task as a measure of short-term memory, which young children can perform reliably, as studies show a high degree of overlap between short-term memory and working memory (see Aben et al., 2012). Moreover, short-term memory has been shown to play a pivotal role in explaining the relationship between working memory and higher reasoning abilities (Hornung et al., 2011). In addition, we administered a task of non-verbal IQ to determine if fine motor skills continue to contribute to early reading and maths ability after controlling for non-verbal IQ (see Roebers et al., 2014).

## Study 1

### Methods

#### Participants

In total, 62 typically developing children attending Year 1 of primary school were recruited from three primary schools located in low SES areas within Nottingham. The sample consisted of 29 males and 33 females that ranged in age between 65 and 80 months (5 years 5 months to 6 years 8 months). None of the children had been identified with special educational needs, indicating the absence of significant motor, intellectual, attentional or behavioral difficulties in this sample.

Ethical approval for the study was granted from the School of Psychology, University of Nottingham, which complies with the ethical guidelines of the British Psychological Society. Informed consent was obtained from parents/guardians for each child who participated in the study.

#### Measures

Pupils were evaluated on fine motor skills and two measures of early scholastic achievement, namely reading and maths. The standardized tests described below were chosen because they are suitable for the age range of pupils in this study and are considered to be ‘gold standard’ assessments of motor skill (Gwynne and Blick, 2004) and have UK norms for reading and maths ability. In addition, a UK based measure of SES was obtained for each pupil.

##### Fine motor skills

The *Bruininks–Oseretsky Test of Motor Proficiency, Second Edition* (BOT-2; Bruininks and Bruininks, 2005) was used to assess fine motor skills. This age-adjusted measure is suitable for children aged 4–21 years and consists of eight subtests. Both subtests of the *Fine Manual Control* composite index were administered. (1) Fine Motor Precision requires children to draw, fold, and cut within a specific boundary, and (2) Fine Motor Integration requires children to reproduce drawings of various geometric shapes that range in complexity from a simple circle to overlapping pencils. Both of these tasks involve activities that require precise control of finger and hand movement. As emphasis is placed on precision of response items are not timed. A composite measure of Fine Manual control was also obtained using the test norms. As reported in the test manual, reliability coefficients (internal consistency) for these two subtests in children aged 5–6 years were high in the normative sample, ranging from 0.75 to 0.84 (Bruininks and Bruininks, 2005, p. 52). For each subtest, items were scored according to the procedure provided in the test manual and standardized scores were generated from raw scores with the gender-specific test norm μ = 15 and σ = 5.

##### Scholastic skills

Early scholastic achievement was evaluated using the *Wechsler Individual Achievement Test, Second Edition* (WIAT-II^UK^; Wechsler, 2005). This age-adjusted measure is suitable for children aged 4–21 years and consists of nine subtests. Two subtests were used in this study. (1) Word Reading which assesses the ability to name single letters, recognize sounds in a word and read whole words, and (2) Mathematical Reasoning which assesses the ability to solve problems about numbers and probability and interpret graphs. As reported in the test manual, reliability coefficients (inter-item comparison) for these two subtests in children aged 5–6 years were high in the normative sample, ranging from 0.92 to 0.99 (Wechsler, 2005, p. 86). For each subtest standardized scores were generated from raw scores with the test norm μ = 100 and σ = 15.

#### Socio-economic Status (SES)

The Income Deprivation Affecting Children Index (IDACI) rank 2010 was used to determine a measure of SES for each pupil. This measure is based on residential postcodes in the UK and reflects the proportion of children aged 0–15 years living in low-income families within a particular postcode area (Department for Communities and Local Government, 2011). On a national scale, rank 1 represents the most deprived area and 32482 represents the least deprived. In this study, IDACI ranks ranged from 56 to 24688, with a median value of 4025 (*n* = 60). SES data was missing for two children so the SES index of their school was used as a close estimate for their SES.

#### Procedure

In each of the three participating schools, Year 1 teaching assistants were trained by the first author to administer the standardized tests described above. A half-day training session was held for the teaching assistants and clear printed guidelines were provided for test administration in addition to the test manuals. Teaching assistants could contact the first author throughout the data collection period with any queries about test administration. Completed response forms were stored securely in each school and were collected by the first author for analysis once data collection was finalized.

Teaching assistants administered each of the four measures described above to individual pupils on a one-to-one basis, in a quiet area of their school, free from distraction. Tests were administered over one or two short sessions, each lasting 10–20 min. Breaks were given in between tests and when necessary in accordance with the child’s engagement with the process. The following fixed order was used to administer the tests: (1) Fine Motor Precision, (2) Fine Motor Integration, (3) Word Reading, and (4) Mathematical Reasoning.

Upon completion of data collection by all three schools, one coder (second author) scored all of the data for every participant on each of the four subtests. The coder received specific training from the first author. As scoring for the motor subtests involved some level of subjective interpretation, a second rater scored a random sample of data from 20 pupils and the agreement between the two raters was calculated using a two-way, mixed, absolute agreement, single-measure intra-class correlation (ICC). The resulting ICC was in the excellent range (ICC = 0.996), indicating high degree of agreement between coders. The second author entered all of the data into SPSS version 22.0 (IBM Corp, 2013) for statistical analysis.

#### Statistical Analysis

For each participant, standard scores were generated for the four subtests and used for all the analyses described below. Normality and equality of variance were explored across the whole sample and across gender and assumptions for parametric statistics were met. Three pupils with relatively high SES were identified as outliers through graphical representation (see Appendix 1) so the same analyses were conducted after removal of these children. As the significance of results did not change following removal of these outliers, results, and figures are reported for the total sample.

Relationships between fine motor, reading, and maths skills were investigated using paired-samples *t*-tests and effect sizes were calculated with Cohen’s *d* (Cohen, 1988). Pearson’s correlations were conducted to explore associations between tasks and Spearman’s rank correlation was used to assess if SES impacted on task performance. For the correlational analyses Bonferroni corrected *p*-values were applied to account for multiple comparisons. The effect of gender on fine motor skills and scholastic attainment was investigated using separate two-way mixed ANOVAs. Finally, the extent to which fine motor skills predicted attainment in reading and maths was explored in two hierarchical multiple regressions. In the final step of each regression analysis, we entered the other scholastic skill to evaluate the unique contribution of fine motor skills whilst controlling for a range of generic skills that contribute to scholastic progression (such as verbal IQ and executive functions). Preliminary analyses ensured no violation of the assumptions of multicollinearity.

### Results

A complete dataset was obtained for 60 pupils. All the pupils completed the two subtests of fine motor skill, one pupil was not given the Mathematical Reasoning subtest and the Word Reading scores for two pupils were excluded because of a mistake in the test administration.

#### Relationships between Fine Motor and Scholastic Skills

Group performance across the four subtests is summarized in **Table 1**. As can be seen, overall group mean scores fall within 1 SD of the test norms for all tasks and the SDs are close to the test norms. Whilst mean performance on the two subtests of fine motor skill was similar overall, there was a noticeable discrepancy in the overall sample between reading and maths of 8.90. This value is below the minimum difference of 9.71 required for statistical significance at 0.05 level of the standardization sample. However, for this sample, a paired-samples *t*-test demonstrated that the overall group difference in reading and maths ability was statistically significant [*t*(59) = 4.71, *p* < 0.001] and was captured by a medium effect size (Cohen’s *d* = 0.58), showing the relative strength for reading.

**Table 1:** Study 1.

Group	Fine Motor Skills (BOT-2)		Scholastic skills (WIAT-II)	
	Mean (*SD*) Min–Max		Mean (*SD*) Min–Max	
	Fine Motor Precision	Fine Motor Integration	Word Reading	Mathematical Reasoning
Boys (*n* = 28)	14.8 (4.6) 6–23	14.3 (5.2) 5–25	102.5 (17.4) 55–130	96.3 (17.2) 63–133
Girls (*n* = 32)	13.6 (4.3) 5–24	13.3 (4.4) 4–22	106.5 (14.7) 83–141	95.3 (12.5) 63–114
Total (*n* = 60)	14.2 (4.4) 5–24	13.8 (4.8) 4–25	104.6 (16.0) 55–141	95.7 (14.7) 63–133

*Group performance (standard score) on each of the four subtests. BOT-2 test norm μ = 15 and σ = 5. WIAT-II test norm μ = 100 and σ = 15.*

A series of Pearson’s correlations were conducted to investigate associations between measures. Results are reported in **Table 2** and scatter plots are available in Appendix I. **Table 2** shows that, as expected, the two measures of fine motor skill correlated significantly as did the two measures of scholastic attainment. Medium to strong positive correlations were also found between fine motor and scholastic skills, and these were significant in all cases except for Word Reading and Fine Motor Precision.

**Table 2:** Study 1.

	IDACI rank	Word Reading	Mathematical	Fine Motor	Fine Motor
			Reasoning	Precision	Integration
**IDACI rank**	/				
Word Reading	ρ = 0.098 *p* = 0.458	/			
Mathematical Reasoning	ρ = 0.186 *p* = 0.155	***r* = 0.550^∗^** ***p* < 0.001**	/		
Fine Motor Precision	ρ = -0.006 *p* = 0.963	*r* = 0.198 *p* = 0.129	***r* = 0.597^∗^** ***p* < 0.001**	/	
Fine Motor Integration	ρ = -0.074 *p* = 0.576	***r* = 0.377^∗^** ***p* = 0.003**	***r* = 0.569^∗^** ***p* < 0.001**	***r* = 0.609^∗^** ***p* < 0.001**	/

Correlations among predictors and outcome variables of Study 1. Spearman’s rank (ρ) and Pearson’s (r) correlation coefficients are reported as appropriate. Significant correlations with uncorrected p-values highlighted in bold. *Significant correlations which survived Bonferroni correction at α = 0.05/4 = 0.013.

#### Impact of SES on Task Performance

A series of Spearman’s rank-order correlations were conducted to explore the relationship between SES and early fine motor and scholastic skills (see **Table 2** and Appendix I). Results revealed no significant correlation between SES and any of the scholastic and motor tasks.

#### Effects of Gender on Task Performance

**FIGURE 1:**
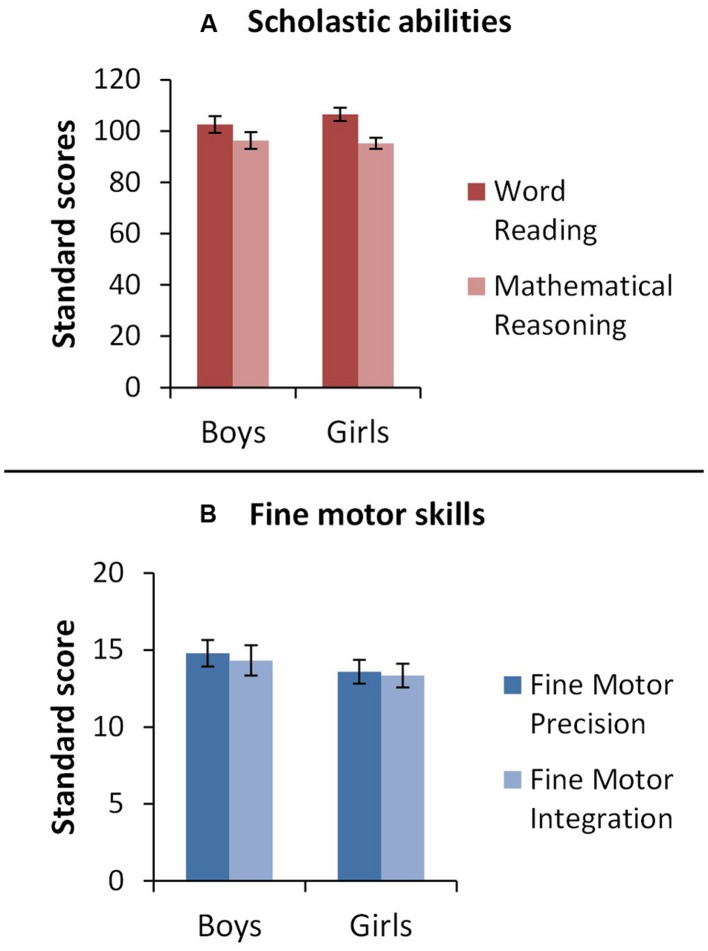
**Study 1.** Bar graph representing mean performance (standard score, *y*-axis) for boys and girls (*x*-axis) for **(A)** scholastic skills and **(B)** fine motor skills. Error bars represent 1 standard error.

To explore the effects of gender on early fine motor skills and scholastic attainment, two separate two-way mixed ANOVAs were conducted with Gender (Boys, Girls) as the between-subjects variable and Task (1: Fine Motor Precision, Fine Motor Integration; 2: Word Reading, Mathematical Reasoning) as the within-groups variable. Results are shown in **Figure 1**. No significant effect of gender on task performance was found for either fine motor skills [*F*(1,58) = 1.03, *p* = 0.314] or scholastic attainment [*F*(1,58) = 0.17, *p* = 0.683] and no significant interaction was found between gender and task performance for either domain [1: Gender and Fine Motor Skills: *F*(1,58) = 0.04, *p* = 0.841; 2: Gender and Scholastic Attainment: *F*(1,58) = 1.75, *p* = 0.192]. Main effects of task corroborated the findings of the paired-sample *t*-tests reported above. Whilst both measures of fine motor skill appear to develop side-by-side [*F*(1,58) = 0.45, *p* = 0.505] scholastic attainment was significantly higher for reading than for maths [*F*(1,58) = 21.57, *p* < 0.001].

#### Predictors of Scholastic Attainment

Two separate hierarchical multiple regressions were performed to investigate the unique contribution that fine motor skills made to the prediction of early reading and maths performance. Gender and SES were not entered into the regression as no significant effects were found. Thus, the three variables that were significantly related to reading or maths were entered progressively into the model in the following order: Fine Motor Precision, Fine Motor Integration and Mathematical Reasoning (for the reading regression) and Word Reading (for the maths regression). Results are summarized in **Table 3**.

**Table 3:** Study 1.

Step	Variable(s)	Model		Significance	Change		Unstandardized Coefficients	Standardized Coefficients	Significance
		*R*	*R*^2^	*F* (df), *p*	Δ*R*^2^	Significance Δ*F*	*B*, *SE*	β	*t*, *p*
**Word Reading (SS)**									
1	Fine Motor Precision	0.20	0.04	2.37 (1,58), 0.129	0.04	0.129	0.71, 0.46	0.20	1.54, 0.129
2	Fine Motor Precision + **Fine Motor Integration**	0.38	0.14	4.79 (2,57), **0.012**	0.10	**0.011**	-0.18, 0.56 1.37, 0.52	-0.05 0.41	-0.33, 0.745 2.64, **0.011**
3	*Fine Motor Precision* + Fine Motor Integration + **Mathematical Reasoning**	0.60	0.35	10.21 (3,56), **<0.001**	0.21	**<0.001**	-1.05, 0.53 0.71, 0.48 0.65, 0.15	-0.29 0.21 0.60	-1.98, 0.*053* 1.4 7, 0.147 4.26, **<0.001**
**Mathematical Reasoning (SS)**									
1	**Fine Motor Precision**	0.60	0.36	32.06 (1, 58), **<0.001**	0.36	**<0.001**	1.99, 0.35	0.60	5.66, <**0.001**
2	**Fine Motor Precision +** **Fine Motor Integration**	0.65	0.42	20.94 (2, 57), **<0.001**	0.06	**0.012**	1.32, 0.42 1.01, 0.39	0.40 0.33	3.13, **0.003** 2.59, **0.012**
3	**Fine Motor Precision +** Fine Motor Integration + **Word Reading**	0.75	0.57	24.22 (3,56), **<0.001**	0.15	**<0.001**	1.39, 0.37 0.50, 0.37 0.38, 0.09	0.42 0.16 0.41	3.75, **<0.001** 1.38, 0.175 4.26, **<0.001**

*Model fits for the hierarchical multiple regressions identifying significant predictors of early reading and maths attainment. Significant predictors highlighted in bold*.

For Word Reading, only models 2 and 3 were statistically significant (*F* ≥ 4.79, *p* ≤ 0.012) and explained 14–35% of the variance. Significant improvements to the model were found at step 2 when adding in Fine Motor Integration (Δ*R*^2^ = 0.10, *p* = 0.011) and at step 3 when adding in Mathematical Reasoning (Δ*R*^2^ = 0.21, *p* < 0.001). While Fine Motor Integration was a significant predictor in model 2 (*p* = 0.011), its contribution was no longer significant when Mathematical Reasoning was added at step 3 (*p* = 0.147). Mathematical Reasoning was the only significant predictor in model 3 and accounted of 21% of unique variance, although Fine Motor Precision showed a strong tendency toward significance (*p* = 0.053).

For Mathematical Reasoning, all models were statistically significant (all *F* ≥ 32.06, *p* ≤ 0.001) and explained 36–57% of the variance. Significant improvements to the model were found at every step of the regression analysis (*p* ≤ 0.012). Whilst Fine Motor Precision was a significant predictor in each model (all *p* ≤ 0.003), the contribution of Fine Motor Integration was no longer significant when Word Reading was added into the model at step 3. Fine Motor Precision accounted of 36% of unique variance, Fine Motor Integration added a further 6% of unique variance, and Word Reading added a further 15% of unique variance.

### Discussion

Study 1 examined the nature of concurrent relationships between Fine Motor Precision and Fine Motor Integration, gender, SES and scholastic achievement in children from low SES backgrounds in Year 1 of primary school in the UK.

Across the sample, group performance on the four subtests was close to the test norms. As expected, the two measures of fine motor skill were closely related as were the two measures of scholastic attainment. However, whilst the two motor skills were similarly developed, a significant discrepancy was found between scholastic skills, with performance in reading exceeding that in maths. As the measures of reading and maths were taken from the same battery of scholastic tests direct comparisons in attainment across domains can be drawn as these subtests have been standardized on the same normative sample. Previous research has identified a close relationship between the development of early literacy and numeracy skills (Purpura et al., 2011; Kleemans et al., 2012). In this study, early reading skills were more advanced than early maths skills. This result is consistent with UK national statistics that show a similar pattern at the end of Key Stage 1 when children are aged 7–8 years (Department for Education, 2014). This may be indicative of the effectiveness of the current UK literacy intervention program that starts during the Foundation Year of schooling when children are aged 4–5 years. Our results suggest an early protective effect of the national literacy intervention for supporting the acquisition of reading skills. In contrast, a similar national level structured early intervention approach for mathematics is not currently implemented in the UK.

The gender analysis showed no significant interaction between gender across scholastic skills. However, the reading-maths discrepancy was captured by a large effect size for girls (Cohen’s *d* = 0.82) and by a small–medium effect-size in boys (Cohen’s *d* = 0.36), suggesting that female pupils were more vulnerable to this discrepancy than their male counterparts. Moreover, no gender effect was found for either fine motor task, suggesting that in this sample fine motor skills have developed similarly in boys and girls. Whilst these results are contrary to current literature reviewing gender differences in educational achievement (Cassen and Kingdon, 2007; Strand, 2014) and motor skill development (Morley et al., 2015), the reading-maths discrepancy reported here nevertheless highlights a need to provide additional support for maths education that would benefit all pupils.

The correlational analyses showed no relationship between SES and fine motor skills or scholastic skills, probably due to the narrow range of SES in our sample. However, the purpose of this study was to examine the relationship between fine motor skills and scholastic attainment in pupils from low SES backgrounds so our sample was drawn from low SES areas. Accordingly, we did not expect to find significant relationships between SES and task performance in this study. Future research might explore how SES influences the development of fine motor and scholastic skills and their associations in a sample drawn from a wider range of SES backgrounds.

Interestingly, when investigating the relation between fine motor and scholastic skills, regression analyses showed a stronger influence of fine motor skills in predicting early maths than early reading skills. Specifically, Fine Motor Integration was shown to be a significant predictor for reading but when Mathematical Reasoning was taken into account Fine Motor Integration no longer significantly predicted reading performance. In contrast, both Fine Motor Integration and Fine Motor Precision significantly predicted maths ability, but the contribution of Fine Motor Integration was no longer significant when Word Reading was taken into account. These results suggests some degree of overlap between Fine Motor Integration and Word Reading and Mathematical Reasoning, which might arise from each of these skills drawing to some extent, at least, on visuo-spatial processes. The additional influence of Fine Motor Precision in predicting maths ability supports recent studies that have highlighted the need for precise manual movements in a range of mathematical tests (e.g., Barnhardt et al., 2005; Simms et al., 2016). Overall, these results support the notion that fine motor skills are more intimately related to early maths than early reading ability. This has important implications for potential maths interventions.

However, before firm conclusions can be drawn it is important to recognize the main limitation of this study, namely, that additional cognitive skills that are also related to scholastic achievement, such as non-verbal IQ and verbal STM were not included in this study. Previous studies that have investigated the influence of these cognitive abilities and of fine motor skills on reading and maths performance have provided mixed evidence. As previously highlighted, Cameron et al. (2012) found specific effects of Fine Motor Integration and executive functions on literacy and maths. Becker et al. (2014) found Fine Motor Integration and behavioral self-regulation predicted early maths and literacy ability but that executive functions were additionally related to early literacy skills. In contrast, Carlson et al. (2013) reported a specific role for Fine Motor Integration, but not fine motor coordination, in predicting mathematics ability but neither components of fine manual control contributed significantly to reading ability when SES, gender and IQ were controlled. Finally, as previously noted, Roebers et al. (2014) failed to find a specific role for fine motor skills and non-verbal IQ in predicting mathematics, reading and spelling abilities, once executive functions were accounted for.

In order to address the contradictory evidence in the current literature, we replicated Study 1 with a younger group of children in Foundation Stage 2 who were aged 4–5 years in Study 2 and included measures of non-verbal IQ and verbal STM. Foundation Stage 2 pupils were selected for Study 2 for two reasons. Firstly, this is the first year of compulsory schooling in the UK and we aimed to examine whether the effects of the national policy focus upon phonics and literacy development were evident, even in children who had just started school. Secondly, additional research suggests that the correlates of educational underachievement lie, at least in part, in the child’s experiences and development during the first year of primary school (Melhuish et al., 2008), leading to a focus upon the interrelationship of variables at that age.

## Study 2

### Methods

#### Participants

Thirty-four typically developing children in the Foundation Stage 2 of one primary school from an average SES area of Nottinghamshire, UK were recruited to the study. The sample included 17 males and 17 females that ranged in age between 50 and 61 months (4 years 2 months to 5 years 1 month). The overall SES of this sample was higher than in Study 1 as IDACI ranks ranged from 5920 to 31716, with a median value of 22512 (*n* = 34), although the sample included some children from deprived backgrounds. None of the children had been identified with special educational needs, indicating the absence of significant motor, intellectual, attentional, or behavioral difficulties in this sample.

Ethical approval for the study was granted from the School of Psychology, University of Nottingham, in line with the British Psychological Society ethical guidelines. Prior informed consent was obtained from parents/guardians for each child that participated in the study.

#### Measures

As in Study 1, children were given the Fine Motor Precision and Fine Motor Integration tasks from the BOT-2 (Bruininks and Bruininks, 2005) to assess fine manual control and Word Reading and Mathematical Reasoning from the WIAT-II (Wechsler, 2005) to assess scholastic attainment. Normative scores for the Mathematical Reasoning subtest of the WIAT-II are not available for children of this age range, so to establish the validity of using this subtest with our sample an additional test for maths ability was given that is appropriate for this age range. The additional maths test consisted of 50 items based on the UK national mathematics curriculum for early years. Basic maths skills assessed in this test included counting, understanding and using numbers, simple addition and subtraction and shape, space, and measure recognition (Outhwaite and Pitchford, under review). Items increased in difficulty in line with progression. No discontinuity rule was applied so all questions were administered. IDACI rank scores were determined from the child’s postcode as an indication of their SES. Additional measures of non-verbal IQ and verbal STM were administered.

##### Non-verbal IQ

The Block Design and Symbol Search subtests from the *Wechsler Preschool and Primary Scale of Intelligence-Third Edition* (WPPSI-III; Wechsler, 2003) were used as a measure of non-verbal IQ. These age-adjusted tasks are suitable for children aged 2 years 6 months to 7 years 3 months. The Block Design subtest requires children to reproduce block patterns presented as a constructed model or picture using one or two colored blocks within a specified time. The Symbol Search subtest requires children to identify whether or not a target symbol appears within an array of similar symbols. Children are given a specified time to conduct the task. As reported in the test manual, reliability coefficients (internal consistency) for these two subtests in children aged 4–5 years were high in the normative sample, ranging from 0.76 to 0.85 (Wechsler, 2003, p. 52). For each subtest, standardized scores were generated from raw scores with the test norm μ = 10 and σ = 3. For each child, scores from the two subtests were averaged to produce a composite measure of non-verbal IQ.

##### Verbal short-term memory (STM)

The Number Recall and Word Order subtests from the *Kaufman Assessment Battery for Children Second Edition* (KABC-II; Kaufman and Kaufman, 2004) were used to measure verbal STM. The KABC-II is an age-adjusted measure that is suitable for children aged 3–18 years. Number Recall requires children to verbally repeat a series of one digit, one syllable numbers, presented to them verbally by the experimenter. Word Order requires children to touch a series of common object silhouettes in the same order as was previously presented verbally by the experimenter. As reported in the test manual, reliability coefficients (internal consistency) for these two subtests in children aged 4–5 years were high in the normative sample, ranging from 0.79 to 0.88 (Kaufman and Kaufman, 2004, p. 88). Raw scores were converted to standardized scores with the test norm μ = 10 and σ = 3. For each child, scores on the two subtests were then averaged to give a composite measure of verbal STM.

#### Procedure

Children were assessed individually by the third author on each of the tasks described above in a quiet area, free from distraction, in their familiar school environment. Tests were administered over three short sessions, each lasting 10–20 min. Short breaks were given between tasks to ensure children remained engaged. Tasks were administered in the following order to be consistent with Study 1: Fine Motor Precision, Fine Motor Integration, Word Reading, Mathematical Reasoning, Block Design, Symbol Search, Number Recall, and Word Order. The second author coded performance of the motor tasks, consistent with Study 1. A second coder (the third author) scored the motor tasks from a random sample of six pupils to ensure inter-rater reliability. The ICC analysis revealed a high level of agreement between the coders (ICC = 0.988).

#### Statistical Analysis

Performance on the Mathematical Reasoning subtest of the WIAT-II was strongly correlated with performance on the early years maths test (*r* = 0.69, *p* < 0.001), demonstrating that the Mathematical Reasoning subtest is a valid measure for this age group. Thus, all subsequent analyses are reported for Mathematical Reasoning. To allow for direct comparisons to be made between Word Reading and Mathematical Reasoning, raw scores were converted to percentage correct, and percentage correct was used for all statistical analyses. Normality and equality of variance were explored across the whole sample and across gender and assumptions for parametric statistics were met. The same statistical analyses as in Study 1 were conducted. Group performance across the two measures of fine motor skill and scholastic skill were examined using paired-samples *t*-tests and effect sizes were calculated using Cohen’s *d* (Cohen, 1988). Associations between fine motor skills, non-verbal IQ, verbal STM, and scholastic abilities were explored using Pearson’s correlations whereas associations with SES and task performance were investigated using Spearman’s rank-order correlations. Bonferroni corrected *p*-values were applied to account for multiple comparisons. The effects of gender on fine motor skills and scholastic attainment was investigated using separate two-way mixed ANOVAs. Finally, two hierarchical regression analyses were conducted to explore the relative contributions of the two measures of fine motor skill, non-verbal IQ, verbal STM in reading and maths attainment when taking into account maths and reading ability, respectively. Preliminary analyses ensured no violation of the assumptions of multicollinearity.

### Results

#### Relationships between Fine Motor Skills, Non-verbal IQ, Verbal STM, and Scholastic Attainment

**Table 4** summarizes group mean performance on the six different ability measures. For the subtests where standardized scores are available, overall group mean performance falls within 1 SD of the test norms for all tasks and the SDs are close to the test norms. Performance on the two subtests of fine motor skill were similar and a paired-sample *t*-test revealed no significant difference [*t*(33) = 0.63, *p* = 0.531]. In contrast, for scholastic abilities a paired-sample *t*-test revealed significantly higher performance in reading than for maths, [*t*(33) = 2.47, *p* = 0.019], with a small-medium effect size (Cohen’s *d* = 0.45).

**Table 4:** Study 2.

Group	Scholastic Skills (WIAT-II)		Fine Motor Skills (BOT-2)		Non-Verbal IQ (WPPSI-III)	Verbal STM (K-ABC)
	*M* (*SD*) Min–Max		*M* (*SD*) Min–Max		*M* (*SD*) Min–Max	*M* (*SD*) Min–Max
	Word Reading	Mathematical Reasoning	Fine Motor Precision	Fine Motor Integration	Block Design and Processing Speed Composite	Word Order and Number Recall Composite
Boys (*n* = 17)	18.1 (9.5) 3.1–32.1	16.9 (3.9) 11.9–25.4	40.3 (11.8) 14.6–61.0	39.0 (16.6) 17.5–72.5	41.6 (12.4) 13.8–65.0	37.5 (7.7) 22.6–49.1
Girls (*n* = 17)	22.5 (9.0) 7.6–35.1	17.1 (4.7) 10.5–30.0	44.5 (11.9) 22.0–68.3	42.5 (19.2) 7.5–67.5	41.1 (8.0) 26.0–53.0	41.2 (8.8) 28.3–54.7
Total (*n* = 34)	20.3 (9.4) 3.1–35.1	17.0 (4.3) 10.5–29.9	42.4 (11.8) 14.6–68.3	40.7 (17.8) 7.5–72.5	41.3 (10.6) 13.8–65.0	39.3 (8.4) 22.6–54.7

*Group performance (% correct) on each of the four subtests*.

A series of Pearson’s correlations was conducted to investigate the associations between the fine motor skills, non-verbal IQ, verbal STM, and scholastic ability. Results are reported in **Table 5** and scatter plots are available in Appendix II. Again, as shown in Study 1, the two measures of fine motor skill correlated significantly as did the two measures of scholastic attainment. For Word Reading, significant, medium-to-strong, positive correlations were found with verbal STM and Fine Motor Integration, although the latter did not survive Bonferroni correction. For Mathematical Reasoning, significant, medium-to-strong, positive correlations were found with Fine Motor Integration and non-verbal IQ, although the correlation with non-verbal IQ did not survive Bonferroni correction. Furthermore, Fine Motor Precision was significantly associated with both non-verbal IQ and verbal STM, whereas Fine Motor Integration was significantly associated only with non-verbal IQ and this correlation did not survive Bonferroni correction.

**Table 5:** Study 2.

	IDACI rank	Word Reading	Mathematical Reasoning	Fine Motor Precision	Fine Motor Integration	Non-verbal IQ	Verbal STM
IDACI rank	/						
Word Reading	**ρ = 0.368** ***p* = 0.032**	/					
Mathematical Reasoning	ρ = 0.073 *p* = 0.682	***r* = 0.584^∗^** ***p* < 0.001**	/				
Fine Motor Precision	ρ = -0.023 *p* = 0.896	*r* = 0.238 *p* = 0.175	*r* = 0.313 *p* = 0.071	/			
Fine Motor Integration	ρ = 0.109 *p* = 0.540	***r* = 0.420** ***p* = 0.013**	***r* = 0.496^∗^** ***p* = 0.003**	***r* = 0.528^∗^** ***p* = 0.001**	/		
Non-verbal IQ	ρ = -0.031 *p* = 0.861	*r* = 0.278 *p* = 0.112	***r* = 0.426** ***p* = 0.012**	***r* = 0.455^∗^** ***p* = 0.007**	***r* = 0.421** ***p* = 0.013**	/	
Verbal STM	ρ = 0.284 *p* = 0.103	***r* = 0.538^∗^** ***p* = 0.001**	*r* = 0.208 *p* = 0.237	***r* = 0.451^∗^** ***p* = 0.007**	*r* = 0.272 *p* = 0.119	*r* = 0.287 *p* = 0.100	/

Correlations among predictors and outcome variables of Study 2. Spearman’s (ρ) and Pearson’s (r) correlation coefficients are reported as appropriate. Significant correlations with uncorrected p-values highlighted in bold. *Significant correlations which survived Bonferroni correction at α = 0.05/6 = 0.008.

#### Impact of SES on Fine Motor and Scholastic Skills

A series of Spearman’s rank-order correlations were conducted to explore the relationship between SES and early fine motor and scholastic skills (see **Table 5**). Results revealed only a weak positive correlation between SES and Word Reading, which did not survive Bonferroni correction.

#### Effects of Gender on Task Performance

**FIGURE 2:**
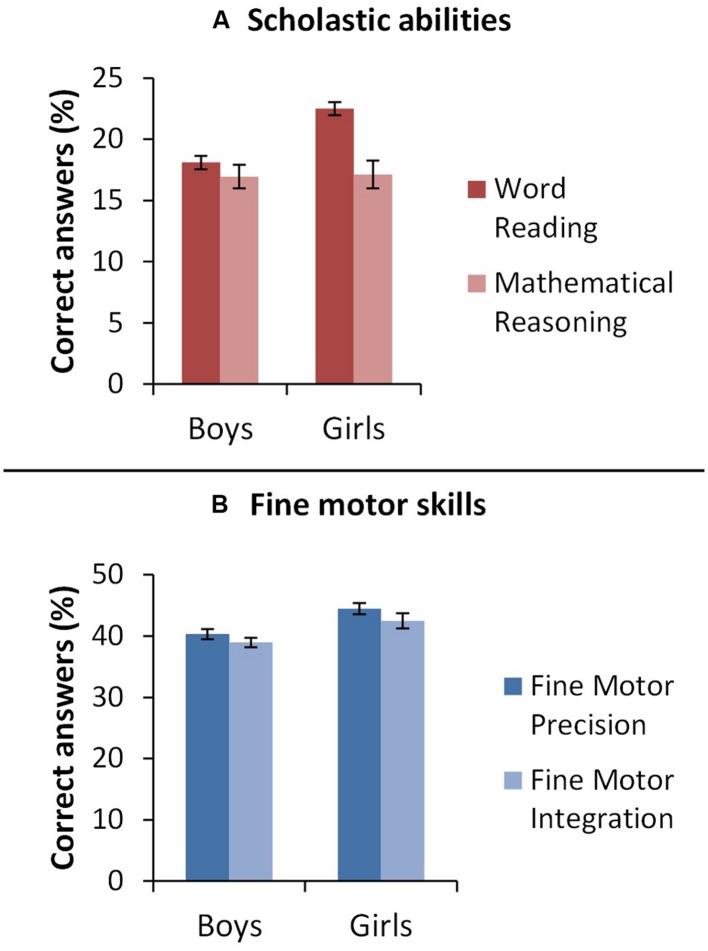
**Study 2.** Bar graph representing mean performance (percentage of correct answer, *y*-axis) for boys and girls (*x*-axis) on **(A)** scholastic skills and **(B)** fine motor skills. Error bars represent 1 standard error.

To explore the effects of gender on early fine motor skills and scholastic attainment, two separate two-way mixed ANOVAs were conducted with Gender (Boys, Girls) as the between-subjects variable and Task (1: Fine Motor Precision, Fine Motor Integration; and 2: Word Reading, Mathematical Reasoning) as the within-groups variable. Results are shown in **Figure 2**. No significant effect of gender on task performance was found for either fine motor skills [*F*(1,32) = 0.74, *p* = 0.395] or scholastic attainment [*F*(1,32) = 1.17, *p* = 0.288] and no significant interaction between gender and task performance was found for either domain [1: Gender and Fine Motor Skills: *F*(1,32) = 0.01, *p* = 0.906; 2: Gender and Scholastic Attainment: *F*(1,32) = 2.68, *p* = 0.112]. Main effects of task corroborated the findings reported in Section “Relationships between Fine Motor Skills, Non-verbal IQ, Verbal STM, and Scholastic Attainment.” Whilst there was no significant difference between group performance on the tasks of fine motor skill [*F*(1,32) = 0.39, *p* = 0.537] scholastic attainment was significantly higher for reading than for maths [*F*(1,32) = 6.39, *p* = 0.017].

#### Predictors of Scholastic Attainment

Two separate hierarchical multiple regressions were conducted to examine the unique contribution of fine motor skills, non-verbal IQ, and verbal STM on early reading and maths ability. Thus, the three variables that were significantly related to reading or maths (prior to Bonferroni correction) were entered progressively into the model in the following order: verbal STM, Fine Motor Integration and Mathematical Reasoning for Word Reading, and non-verbal IQ, Fine Motor Integration and Word Reading for Mathematical Reasoning. Results are summarized in **Table 6**.

**Table 6:** Study 2.

Step	Variable(s)	Model		Significance	Change		Unstandardized Coefficients	Standardized Coefficients	Significance
		*R*	*R*^2^	*F* (df), *p*	Δ*R*^2^	Significance Δ*F*	*B*, *SE*	β	*t*, *p*
**Word Reading (%)**									
1	**Verbal STM**	0.54	0.30	13.40 (1,32), **0.001**	0.30	**0.001**	0.61, 0.17	0.54	3.66, **0.001**
2	**Verbal STM** + Fine Motor Integration	0.61	0.38	9.19 (2,31), **0.001**	0.08	0.060	0.52, 0.17 0.15, 0.08	0.46 0.29	3.11, **0.004** 1.95, 0.060
3	**Verbal STM** + Fine Motor Integration + **Mathematical Reasoning**	0.73	0.53	11.23 (3,30), **<0.001**	0.16	**0.004**	0.48, 0.15 0.04, 0.08 1.0, 0.32	0.43 0.07 0.46	3.24, **0.003** 0.49, 0.627 3.16, **0.004**
**Mathematical Reasoning (%)**									
1	**Non-verbal IQ**	0.42	0.18	6.79 (1,32), **0.014**	0.18	**0.014**	0.25, 0.10	0.42	2.61, **0.014**
2	Non-verbal IQ + **Fine Motor Integration**	0.55	0.30	6.69 (2,31), **0.004**	0.13	**0.024**	0.16, 0.10 0.16, 0.07	0.26 0.39	1.57, 0.127 2.37, **0.024**
3	Non-verbal IQ + Fine Motor Integration + **Word Reading**	0.68	0.46	8.37 (3,30), **<0.001**	0.15	**0.007**	0.13, 0.09 0.09, 0.06 0.10, 0.04	0.21 0.23 0.44	1.44, 0.159 1.42, 0.165 2.91, **0.007**

*Model fits for the hierarchical multiple regressions identifying significant predictors of early reading and maths attainment in Study 2. Significant predictors highlighted in bold.*

For Word Reading, all models were statistically significant (*F* ≥ 9.19, *p* ≤ 0.001) and explained a total variance ranging from 30 to 53%. Significant improvements to the model were found at steps 1 and 3 but not at step 2 when Fine Motor Integration was added to the model (Δ*R*^2^ = 0.08, *p* = 0.060). Verbal STM was found to be a significant contributor at all steps and accounted for a unique 30% of total variance. On the contrary, Fine Motor Integration was not a significant predictor at either step 2 or 3. Finally, Mathematical Reasoning was found to be significant contributor to Word Reading at step 3 and accounted for 16% of total variance.

For Mathematical Reasoning all models were statistically significant (all *F* ≥ 6.69, *p* ≤ 0.014) and explained 18–46% of the total variance. Significant improvements were reported at each step of the regression (all Δ*R*^2^ ≥ 0.13, *p* ≤ 0.024). At each step, the last variable entered became the only significant predictor of the model. Thus, significant predictors were non-verbal IQ only at step 1, Fine Motor Integration only at step 2, and Word Reading only at step 3 (see **Table 6**).

### Discussion

Consistent with Study 1, performance on all tasks was close to the test norms thus this sample can be considered representative of a wider population. Again, whilst no difference was found between the two fine motor tasks, a significant advantage for reading in comparison to maths was revealed (Cohen’s *d* = 0.45). It is interesting that the difference between reading and maths was evident with this group of 4- to 5-year-old children, as they had only just started primary school. This replicates the findings from Study 1, where the reading-maths discrepancy was captured by a larger effect size of 0.58. Overall, these results suggest that the discrepancy between early reading and maths ability is present at the start of primary school and tends to increase over the first 12 months.

There are several reasons why this discrepancy in reading and maths ability is shown in children that have just started compulsory schooling in the UK. It could be that the test of Mathematical Reasoning is not as sensitive as the Word Reading subtest for children aged 4–5 years. Although normative data is not available from the WIAT-II for Mathematical Reasoning for children aged 4–5 years, we showed that the performance of pupils in our sample correlated highly with a non-standardized test of maths that is based on the early curriculum in the UK, thus providing validation for its use with this age group. Furthermore, the correlation between percentage correct on Word Reading and Mathematical Reasoning for the 4- to 5-year-old children (*r* = 0.584) was similar to that of the 5- to 6-year-old year children in Study 1 using standard scores (*r* = 0.550), illustrating a similar strength of association between these two measures of scholastic attainment across the first 2 years of primary school. Alternatively, the discrepancy in reading and maths ability that was found in our study might reflect the focus on teaching phonics that is implemented across all UK primary schools in the early years. The relatively higher scores in reading compared to maths suggest that the phonic intervention that is implemented in this age group at a national level has an immediate and sustained positive effect on reading attainment.

Similar to Study 1, the two motor skills were strongly associated, but only Fine Motor Integration correlated significantly with reading and maths ability. This supports our prediction that the influence of fine motor precision on reading and maths ability may become stronger over the first year of schooling, as children practice scripting letters and numbers and linking these symbolic representations to the underlying phonological and numerical concepts. Furthermore, verbal STM was associated with early reading ability whereas non-verbal IQ was associated with early maths skills. Consistent with Study 1, no gender differences were found, confirming a similar progression for boys and girls in this younger age group.

As with Study 1, the regression analyses showed a different pattern of results for reading and maths. For the prediction of reading performance, verbal STM was identified as a significant predictor at all steps, explaining a unique 30% of the total variance. Fine Motor Integration was no longer a significant predictor of early reading ability once verbal STM and Mathematical Reasoning were accounted for. In contrast, the regression analysis for maths showed that non-verbal IQ, Fine Motor Integration and Word Reading accounted for an equal portion of the total variance explained by the regression model (13–18%). Fine Motor Integration remained a significant predictor of early maths ability once non-verbal IQ was accounted for, but not when Word Reading was added into the model at step 3. The results from step 3 of the regression analyses should be treated with caution, however, due to the likely lack of power in the model when three predictor variables are considered with a relatively small sample size.

Overall, the results from Study 2 corroborate those of Study 1 in that Fine Motor Integration was found to be a significant predictor of early maths ability, but not a significant predictor of early reading ability, even when relevant cognitive abilities were taken into account. Our results are similar to those of Mayes et al. (2009) who found that, above the main contribution of IQ, short-term memory significantly predicted word reading, whereas visuo-spatial integration and grapho-motor ability significantly predicted maths ability in elementary school pupils (kindergarten through fifth grade). Together, these studies indicate an enhanced role for Fine Motor Integration in early maths development compared to early reading development. This has implications for the design of interventions to support the development of early maths skills.

## General Discussion

The role of fine motor skills in early development and subsequent educational achievement has begun to be clarified by recent studies. The two studies reported here present an investigation of these concurrent relationships and of the additional contribution of short-term memory and non-verbal IQ in typically developing, low-mid SES populations. Concurrent relationships were captured between variables at critical points in development leading into early childhood, thereby offering evidence to support the fine-tuning of programs at a pivotal stage for early education. A key finding was that for these groups, attainment was higher in reading than for maths. This contradicts the current national picture and suggests the need for greater consideration of the early years maths curriculum. Furthermore Fine Motor Integration was pinpointed by both studies as a key variable in predicting maths attainment, resilient to effects of cognition. Fine Motor Integration correlated positively with maths performance in both studies, whereas Fine Motor Precision correlated significantly with maths only in Study 1, when children had been in compulsory schooling for one year. Furthermore, in Study 1, Fine Motor Precision remained a significant predictor of maths ability even when the influence of reading acquisition was taken into account. This suggests that the influence of Fine Motor Precision on maths ability emerges over the first year of schooling and might be closely linked with the numeracy skills that children are acquiring over the first year of primary school and the practice children have in writing numbers and carrying out other maths-based activities that require precise motor movements, such as cutting out shapes and using aids such as Snap Cubes^®^ and Numicon.

Several potential mechanisms might underpin the association between fine motor skills and maths ability reported here. For example, Luo et al. (2007) proposed there may be a common window of biological maturation across domains, or that both domains facilitate mental development, or that there might be a common supra-ordinate category of general intelligence, or that the association arises from a stimulating parenting style that cuts across domains. Other authors have given more specific reasons, suggesting that finger counting could be the linking mechanism between fine motor skills and mathematical skills (Di Luca and Pesenti, 2011; Moeller et al., 2011). Finger counting might be closely related to the emergence of Fine Motor Precision becoming a significant predictor of maths ability in Study 1, as children learn to count over the first year of primary school. The absence of a significant correlation between Fine Motor Precision and maths in Study 2, during the first year of compulsory schooling in the UK, suggests an intimate link between the development of fine motor precision and maths skills in response to teaching. Another possibility that could account for the significant correlation between fine motor integration and maths ability found across both age groups (Study 1 and Study 2) is that both fine motor integration and maths ability utilize visuo-spatial processes that are coordinated through a common neural pathway, such as the feed-forward feed-back connections between cerebellum and pre-frontal cortex. When the cerebellum is damaged in early childhood, prior to the onset of formal schooling, strong associations between fine manual control and scholastic abilities, including mathematics, have been found (Davis, unpublished thesis, p. 217). Furthermore, visuo-spatial skills and visuo-motor integration have been recently associated with number line estimation tasks (Simms et al., 2016) which are known to be strong and reliable predictors of mathematical attainment.

The finding that fine motor skills are intimately linked to early maths attainment has implications for educational intervention. Within the UK there are national concerns regarding the persistent low attainment in maths. For example, the UK is currently 26th out of 65 countries for maths attainment by school-leaving age (APPG, 2014). Consequently, concerns are particularly focused upon the educational trajectory of lower SES groups, and upon the importance of early years intervention to reduce the achievement gap as the lower SES groups move through schooling (George et al., 2012). The critical importance of fine-tuning interventions toward appropriate mechanisms to enable progress by the higher risk groups is evident. Key, for example, to the contingency of early years provision in enhancing later outcomes is the quality of provision (Sammons et al., 2004; George et al., 2012), highlighting the need for precision in the nature of the curriculum offered.

Higher educational risk forms through a complex interplay of factors, which require differentiated understanding, at population and community levels (Strand, 2014), and school-level (Sammons et al., 2004; Hargreaves, 2014). School improvement research has included a focus, amongst other features, upon curriculum and pedagogy to explore and mediate the relationships governing scholastic outcomes (Muijs et al., 2004; Strand, 2010). Developmental psychology is well-placed to provide empirically grounded insights into development in informing educational programs, and is arguably underused in the thrust toward school improvement.

The results from our studies challenge previous research (Purpura et al., 2011; Kleemans et al., 2012) and national level data on the relative acquisition of reading and maths (Ofsted, 2015) where both domains in the early years and start of schooling typically appear as commensurate, including for children of lower and average SES households. In accord with the review of educational provision by Ofsted (2015), our results suggests that there may, in contrast to the aggregated picture, be significant local variation in practice and in outcomes within the numeracy curriculum in the early years, undermining the policy drive to overcome factors influencing poorer trajectories for those of lower SES, and the national gap in performance between higher and lower SES groups.

Our results suggest there may be potential risk within the early years curriculum where there is a strong focus on supporting the acquisition of literacy skills through the implementation of a national literacy strategy but no similar strategy for mathematical development. The longer-term consequences of not focussing on early numeracy have been noted (Melhuish et al., 2008) and this concern is reflected at a policy level, in calls for a greater focus upon the teaching of early years maths (APPG, 2014). The greater relative attention paid in the past decade to literacy interventions in the Foundation Stage and Key Stage 1 in the UK has prioritized phonic knowledge and skills in the curriculum (Department for Education, 2014) with comparatively less focus upon the teaching of maths, a phenomenon confirmed by a recent investigation into early years provision and its effects (Ofsted, 2015). Identifying the relatively lower focus upon maths education in early years settings currently, it was noted that this was the view of practitioners themselves, some of whom felt less confidence in the specifics of promoting mathematics in the early years (Ofsted, 2015).

Debate upon the teaching of maths has noted various questions requiring still further empirical evidence, for example: the extent to which mathematical knowledge goals are focused on at the expense of number sense (Dyson et al., 2013); types of instruction (Fuchs et al., 2013); the role of executive functions (Cragg and Gilmore, 2014; Carden and Cline, 2015); the relative role of spatial skills (Nunes et al., 2009; Cheng and Mix, 2014); and more recently, the neural predictors of response to instruction (Supekar et al., 2013). There is, in addition, a long-term debate in early years education on the relative need for discovery or play-based learning versus the role of instruction (Gifford, 2004; Chambers et al., 2010).

Evidence in our studies potentially signals the value of a highly active component of the universal curriculum, in order to promote fine motor skills, with implied gains possible in scholastic achievement. A play-based curriculum could be entirely consonant with one that also holds a focus upon the development of spatial skills such as those employed by Verdine et al. (2014). The need for discrete, although age appropriate instruction, is also underlined through the systematic review evidence of Sharples et al. (2011), which signals the importance of an instructional component in any early years curriculum that seek to reduce the attainment gap for children of lower SES. In addition, the absence of evidence in our studies of significant difference in fine motor skill by gender, in contrast to data elsewhere, consolidates the argument in favor of a universal approach to fine motor skill promotion, as does the report of the current lowering of motor skill norms at a population level (Gaul and Issartel, 2016).

The generic early years curriculum is thought to promote positive motor developments (Marr et al., 2003). Bala et al. (2010), for example, found that a longer period at kindergarten was associated with better grapho-motor skills (fine hand coordination, as well as ability to copy different figures as a whole and their parts) in both females and males. Interestingly, those principalities with higher numeracy outcomes, long term, are generally those where formal education commences later (Jerrim and Choi, 2014). It is likely that, prior to entry into compulsory education, a carefully structured play-based approach in the early years would simultaneously support fine motor skills and the development of number sense, through children’s interactions with objects and their environment.

Finally, the rationale for developing fine motor skills in young children is supported by data illustrating the promotion of cognitive or scholastic gains as dependent outcomes from motor skills interventions. Despite low–weak correlations in the studies considered, the systematic review of evidence by van der Fels et al. (2014) led to a conclusion that fine motor skills interventions might support development in other domains. In concordance with the pattern of data here, Westendorp et al. (2014), working with a learning-disabled population, found evidence of positive effects of a ball skill intervention upon problem-solving skills. Other evidence is available in respect of specific populations, such as those with Developmental Co-ordination Disorder (Bond, 2011), where some positive gains have been found possible through targeted educational intervention, and Case-Smith (1996) showed the effectiveness of an occupational therapy service in preschool children, in relation to fine motor skill and self-care, mobility, and social function.

## Conclusion

The evidence that there can be significant pockets of delay in maths attainment relative to literacy, in low SES groups, and that Fine Motor Integration can be closely related to maths outcomes enhances the argument for a closely focused early years maths curriculum, potentially with a strong enactive and spatial training element, to support visual-motor integration skills.

The extrapolations from this data could be enhanced by further predictive studies, with additional measurement of executive function skills within the population. Because the data here contradicts the national picture upon numeracy attainment, further similar investigations are warranted, to explore whether this finding exists, localized, elsewhere. Intervention studies that offer greater insight into the specific role of Fine Motor Integration and Fine Motor Precision in maths activities, and their contribution to diverse aspects of maths scholastic attainment, would also be welcome. Finally, investigations encompassing older age groups, or longitudinal data, would be valuable, in order to gain insight into how the relationship between Fine Motor Integration and Fine Motor Precision and maths may change with age, together with a greater knowledge of the contribution of other skills, such as executive functions, with the passing of time.

Overall, the results from both of our studies showed relative strengths in reading compared to maths in young pupils from low-to-mid SES backgrounds. Furthermore, both studies showed fine motor skills were not influenced by gender or SES, but were closely related to early maths skills, in particular Fine Motor Integration, even when additional cognitive skills were accounted for. Together, this provides clear evidence for the need for an early intervention approach to maths education, which includes a fine motor skill component, in particular visuo-spatial skills requiring fine motor integration.

## Author Contributions

NP lead the research. She ran Study 1, wrote the methods, results, and discussion, co-wrote the introduction with CP and edited the overall manuscript. CP scored and analyzed the data for studies 1 and 2, co-wrote the introduction with NP, took responsibility for the references and edited the overall manuscript. LO conducted study 2, wrote the methods and edited the overall manuscript. AG informed on the research programme from an educational psychology perspective. She wrote the general discussion and edited the overall manuscript.

## Conflict of Interest Statement

The authors declare that the research was conducted in the absence of any commercial or financial relationships that could be construed as a potential conflict of interest.

## Footnotes

1 See http://www.nottinghamshire.gov.uk/care/childrens-social-care/nottinghamshire-childrens-trust/child-poverty
