# Supplementary material for: Fine Motor Skills Predict Maths Ability Better than They Predict Reading Ability in the Early Primary School Years
Source: Front Psychol. 2016 May 30;7:783. doi: 10.3389/fpsyg.2016.00783 (PMC4884738; doi:10.3389/fpsyg.2016.00783)

**Appendix I**

Study 1. Scatterplots showing the relationships between the variables using standard scores (SS). Pearson’s (r) or Spearman’s (ρ) correlation coefficients are reported as appropriate and flagged with an asterisk if significant following Bonferroni correction. Regression line is displayed for parametric correlations.


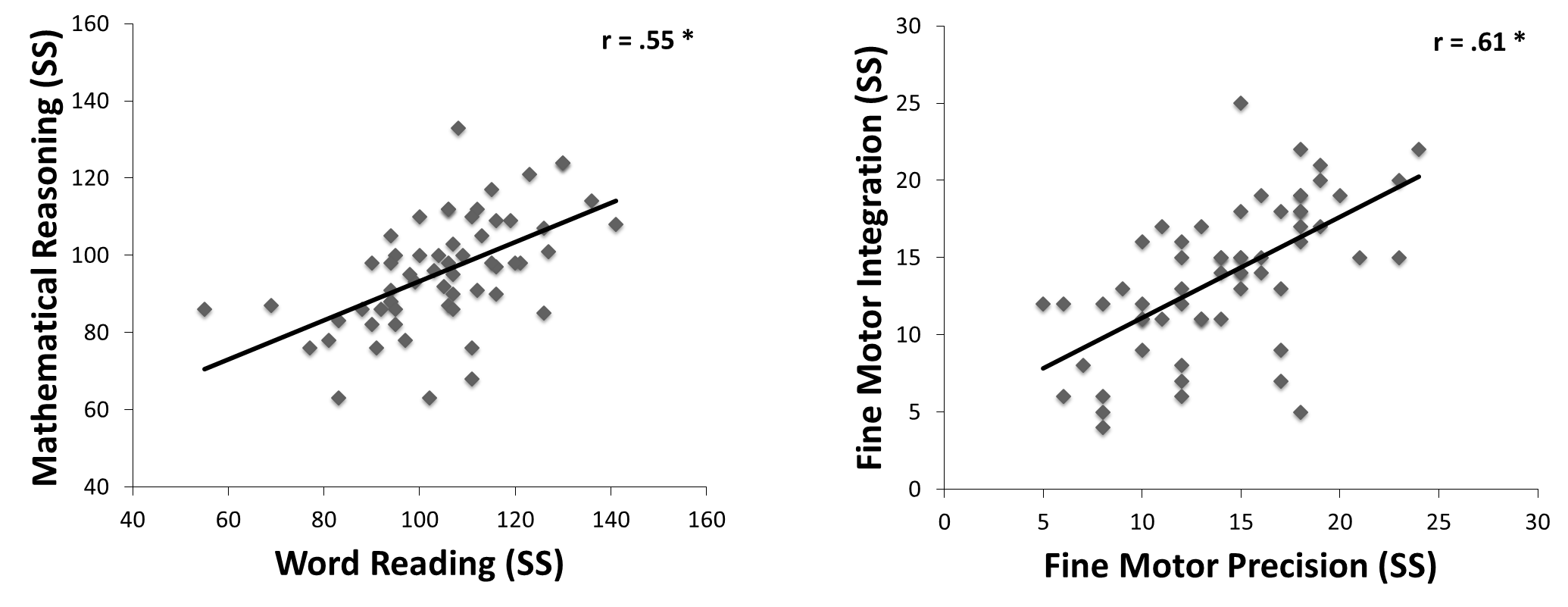


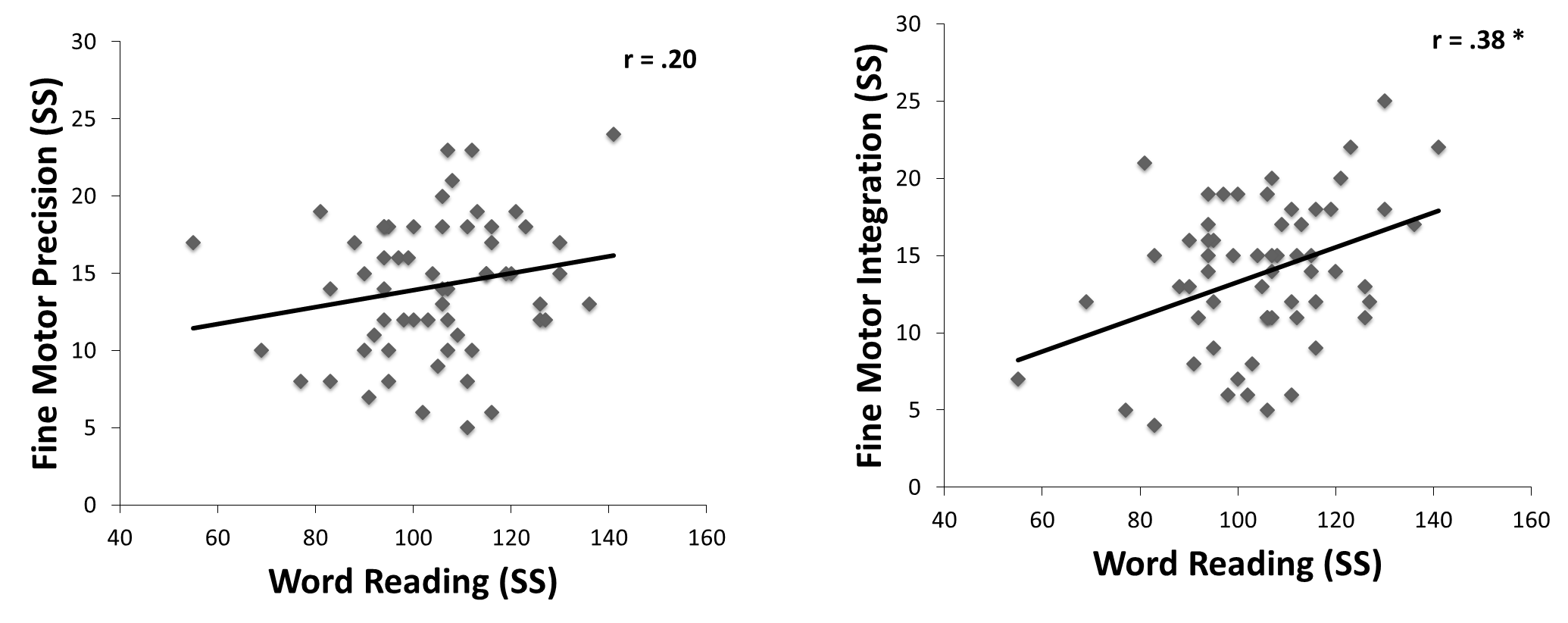


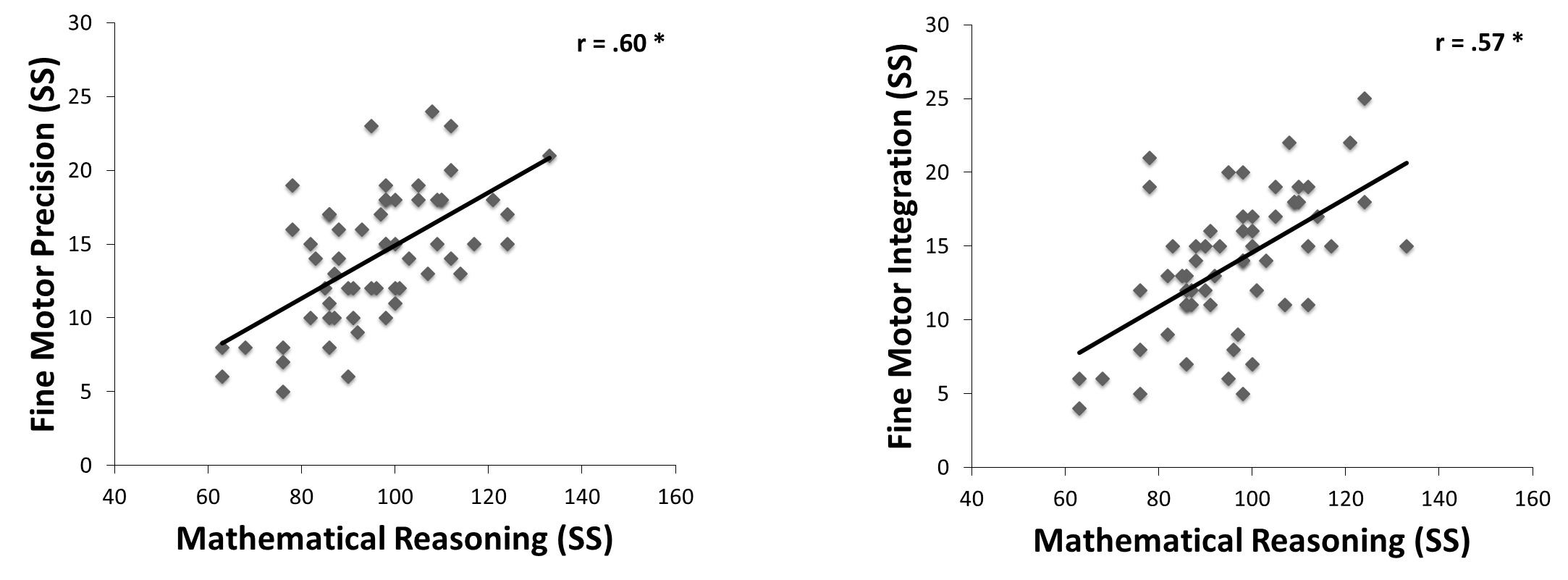


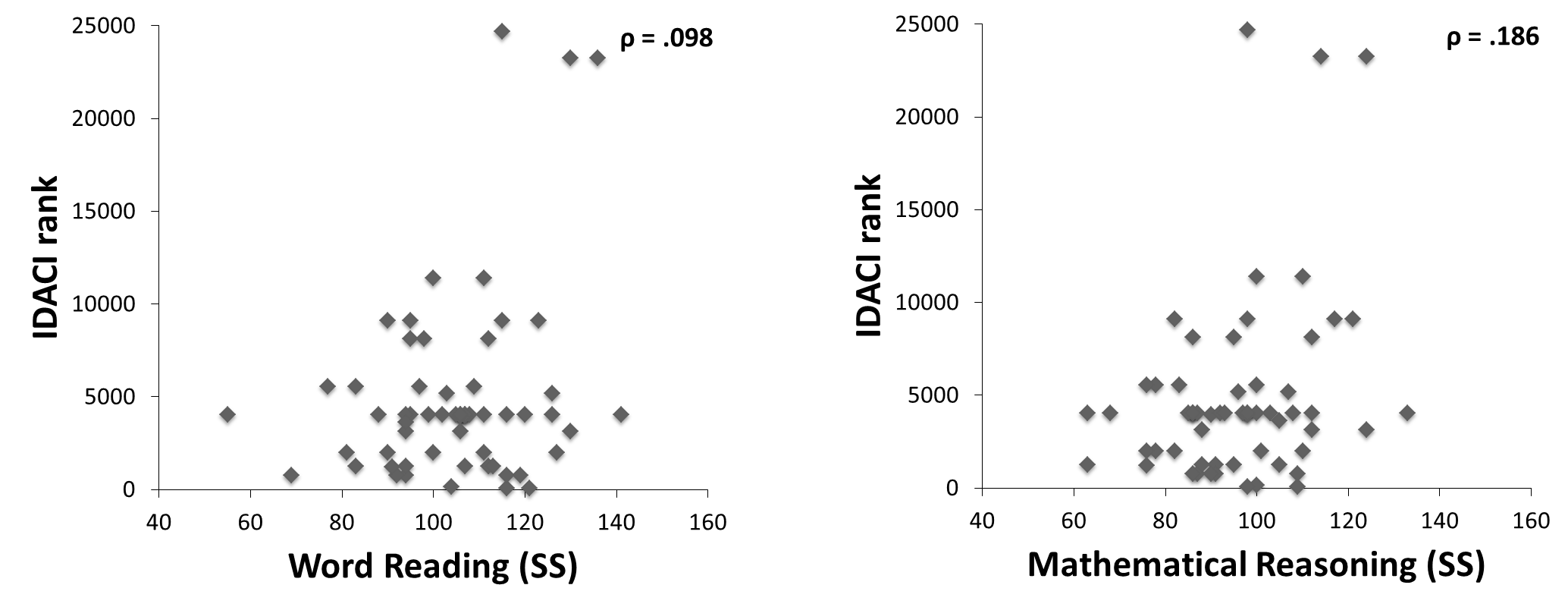


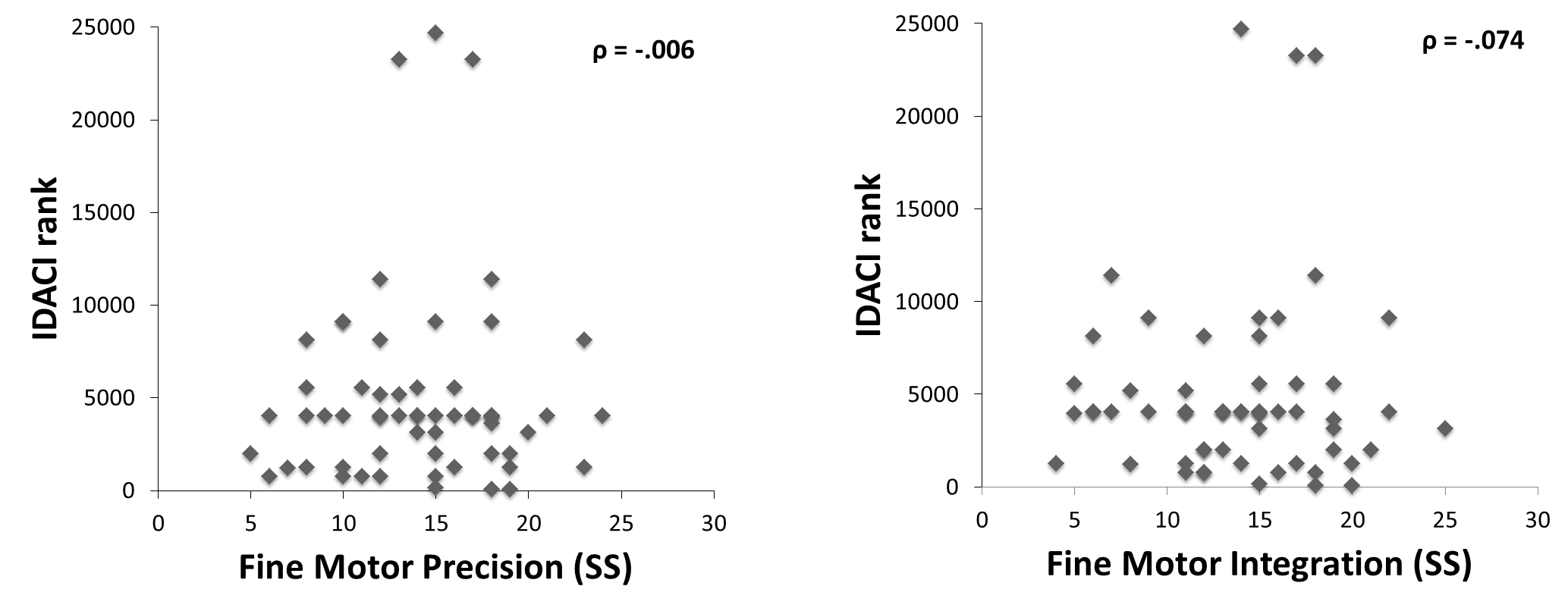


**Appendix II**

Study 2. Scatterplots showing the relationships between the variables using percentage of correct answers (%). Pearson’s (r) or Spearman’s (ρ) correlation coefficients are reported as appropriate and flagged with an asterisk if significant following Bonferroni correction. Regression line is displayed for parametric correlations.


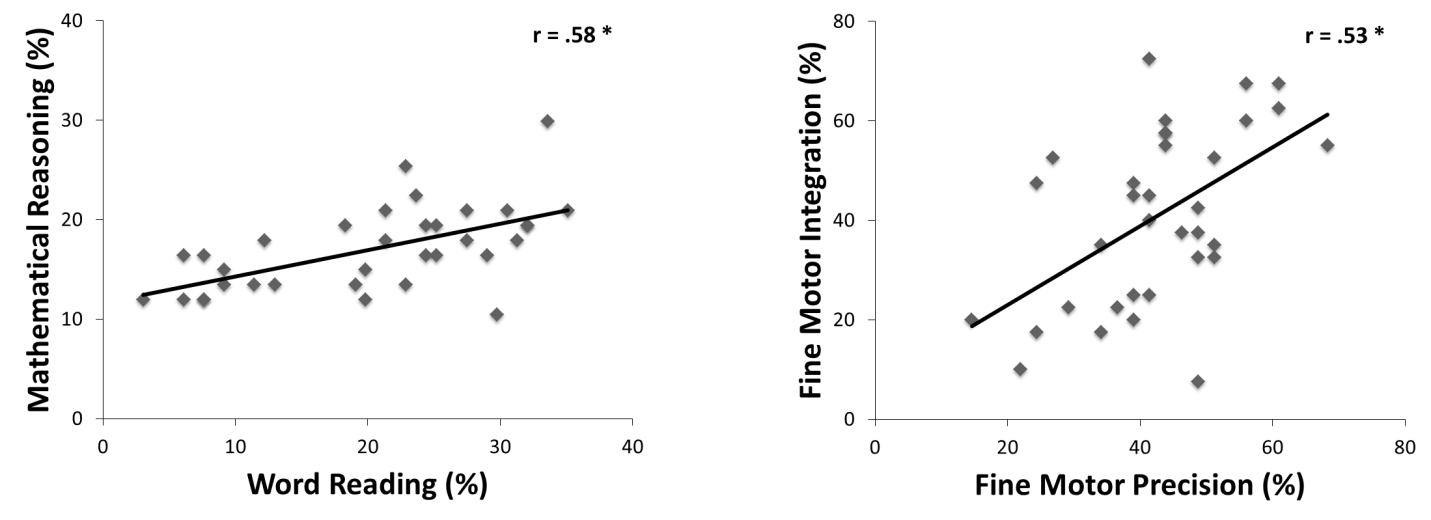


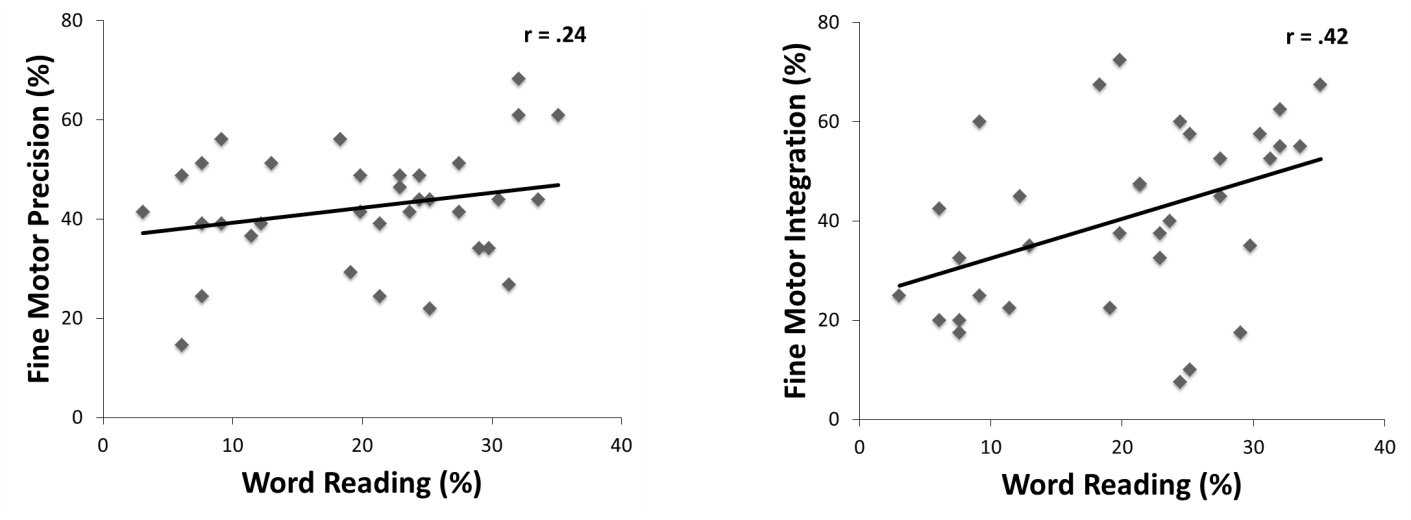


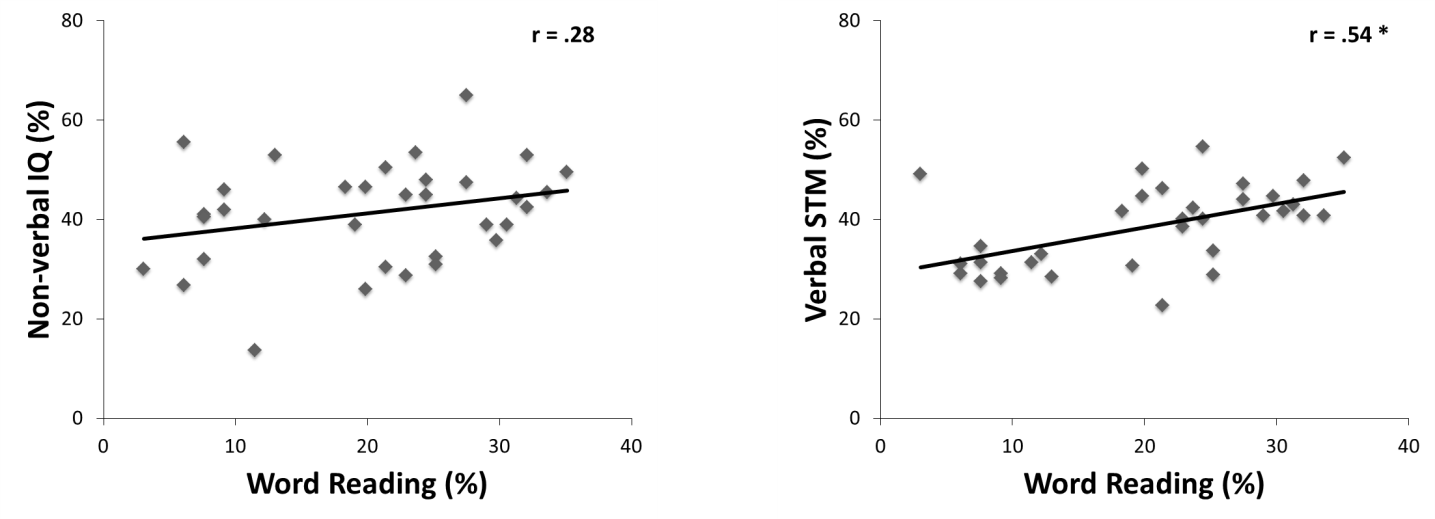


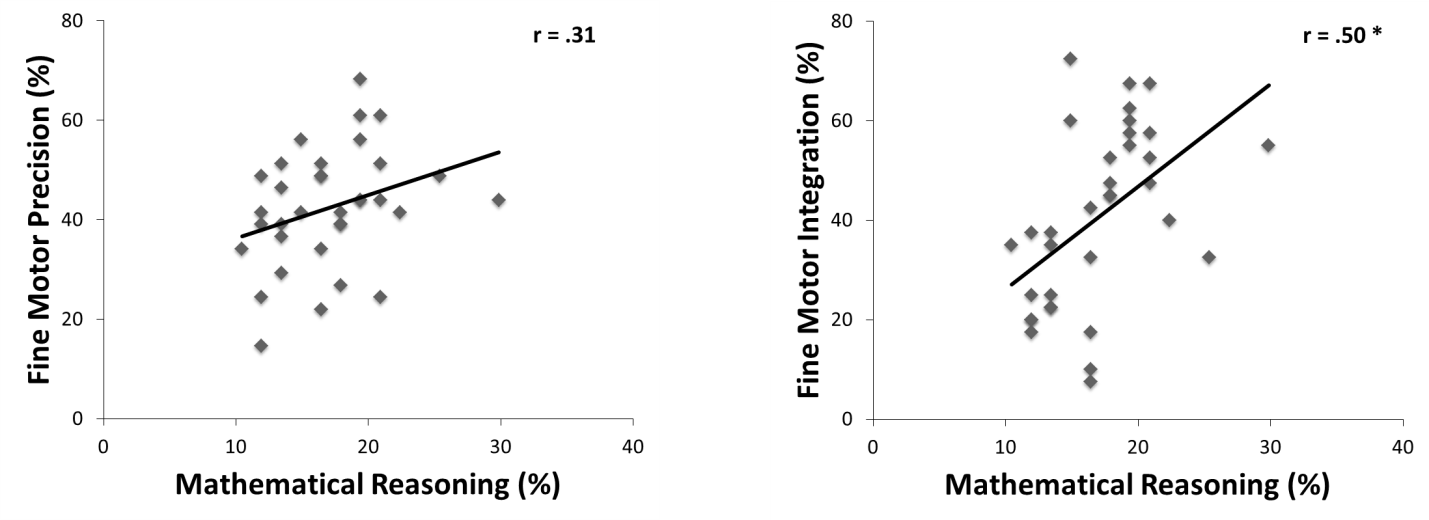


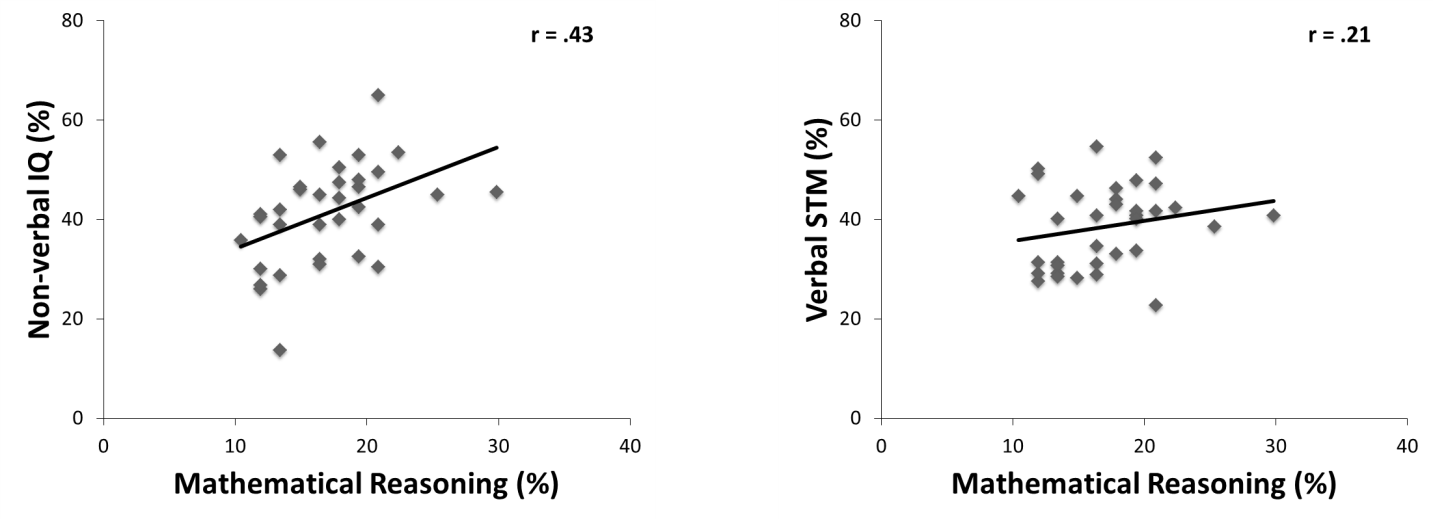


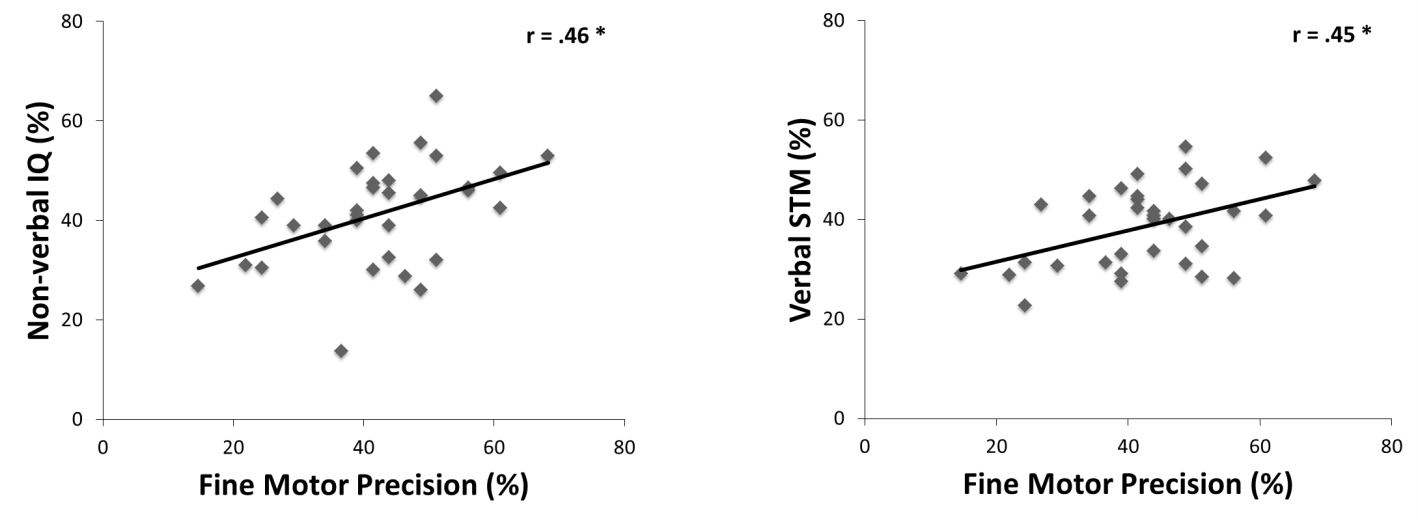


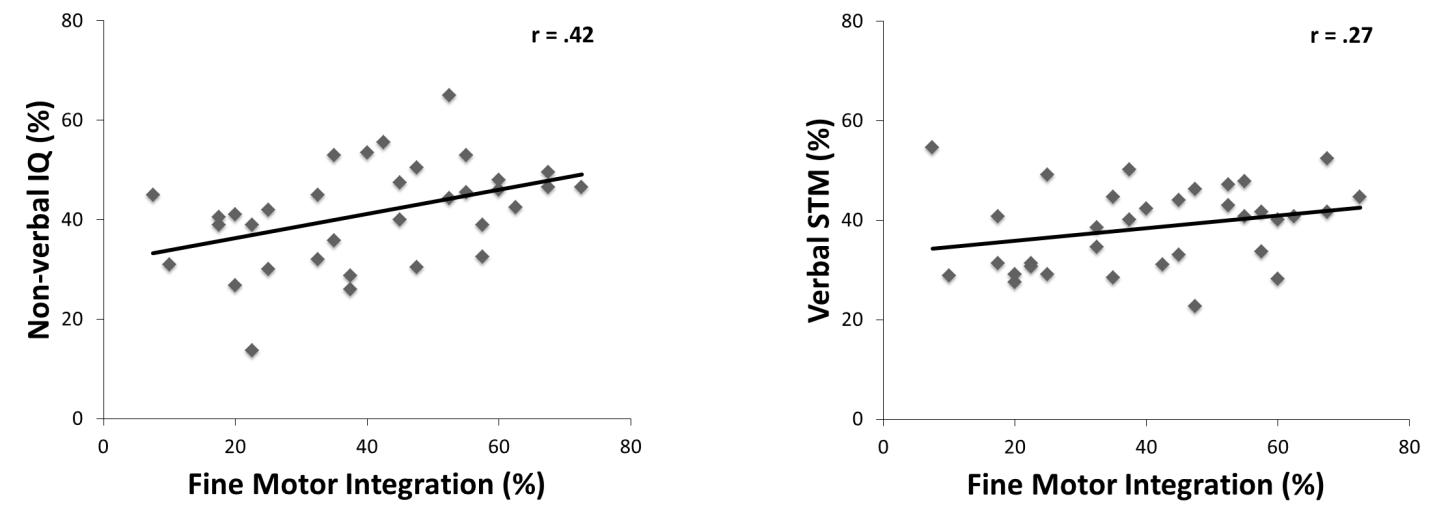


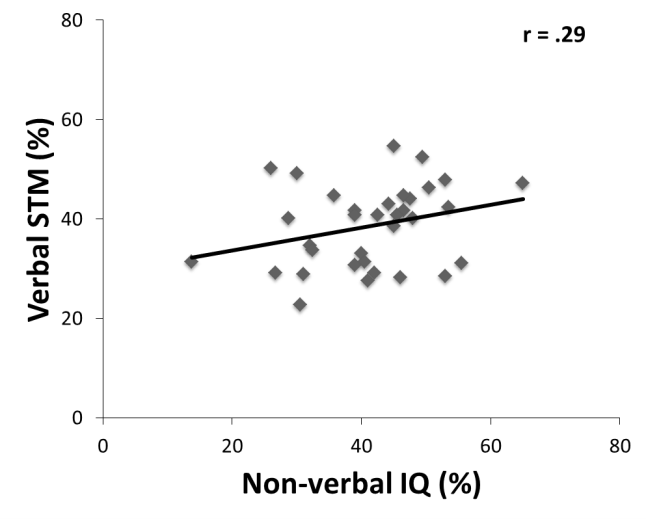


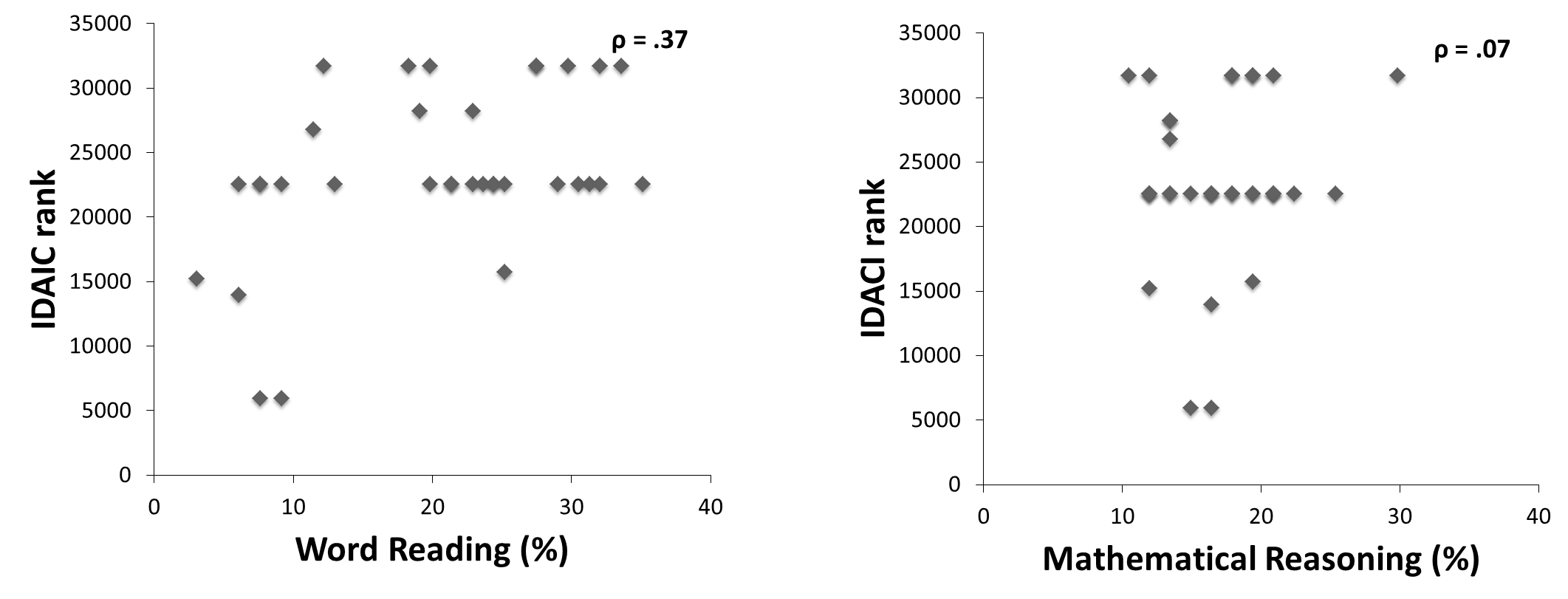


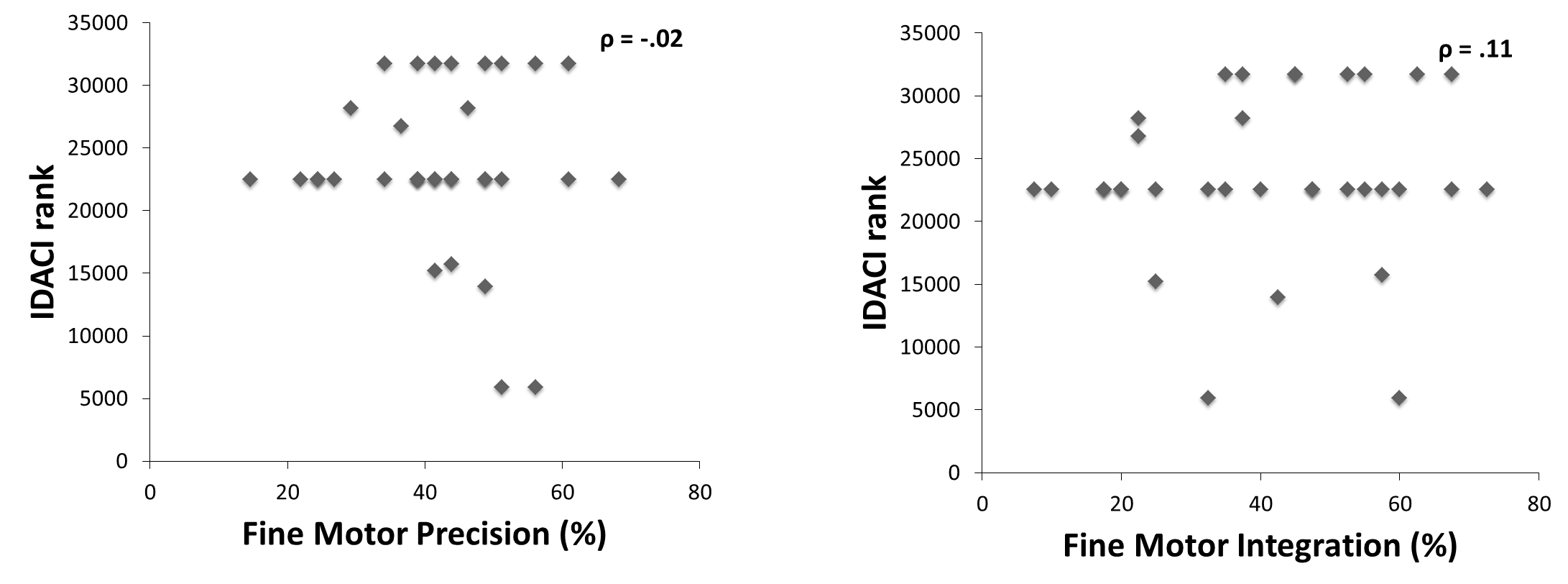


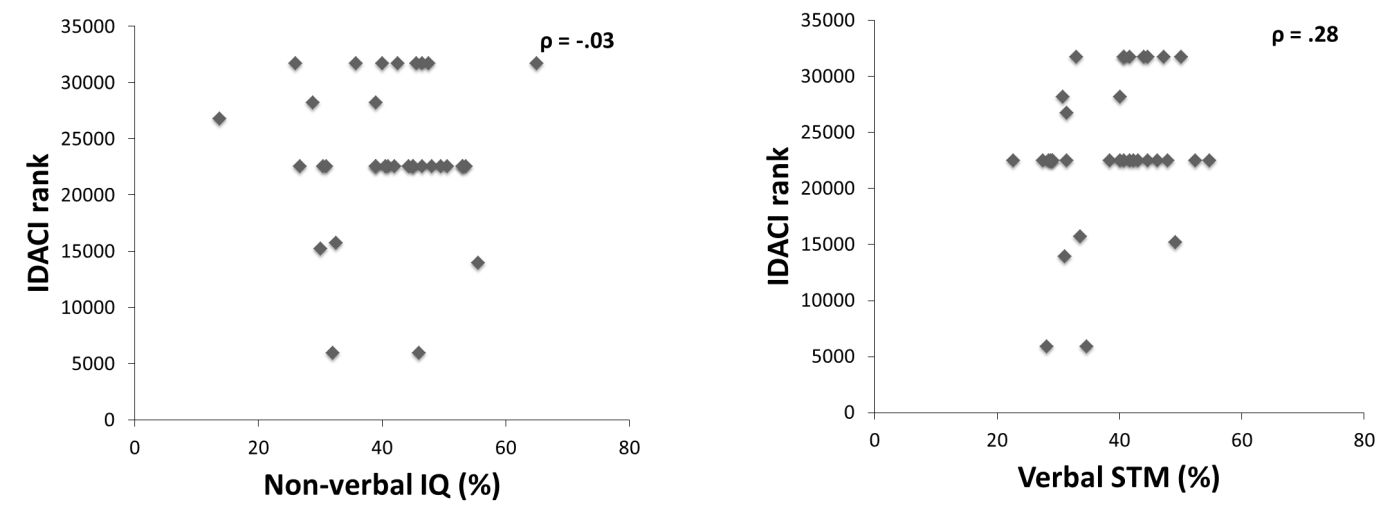

Supplement: Supplementary file 1 [file Data_Sheet_1.DOCX]
